# Hypoxia-induced SKA3 promoted cholangiocarcinoma progression and chemoresistance by enhancing fatty acid synthesis via the regulation of PAR-dependent HIF-1a deubiquitylation

**DOI:** 10.1186/s13046-023-02842-7

**Published:** 2023-10-11

**Authors:** Yananlan Chen, Xiao Xu, Yirui Wang, Yaodong Zhang, Tao Zhou, Wangjie Jiang, Ziyi Wang, Jiang Chang, Shuochen Liu, Ruixiang Chen, Jijun Shan, Jifei Wang, Yuming Wang, Changxian Li, Xiangcheng Li

**Affiliations:** 1 Hepatobiliary Surgery Hepatobiliary Center, The First Affiliated Hospital of Nanjing Medical University, 300 Guangzhou Road, Nanjing, Jiangsu Province China; 2 Key Laoratory for Liver Transplantation, NHC Key Laboratory of Living Donor Liver Transplantation, Chinese Academy of Medical Sciences, Nanjing Medical University), Nanjing, Jiangsu Province China; 3 Wuxi Medical Center, Nanjing Medical University, Wuxi, China

**Keywords:** SKA3, Hypoxia, HIF-1a, Deubiquitylation, Cholangiocarcinoma

## Abstract

**Background:**

Spindle and kinetochore-associated complex subunit 3 (SKA3) plays an important role in cell proliferation by regulating the separation of chromosomes and their division into daughter cells. Previous studies demonstrated that SKA3 was strongly implicated in tumor development and progression. However, the roles of SKA3 in cholangiocarcinoma (CCA) and the underlying mechanisms remain unclear.

**Methods:**

Next-generation sequencing (NGS) was performed with paired CCA tissues and normal adjacent tissues (NATs). SKA3 was chose to be the target gene because of its remarkably upregulation and unknown function in cholangiocarcinoma in TCGA datasets, GSE107943 datasets and our sequencing results. RT-PCR and immunohistochemistry staining were used to detect the expression of SKA3 in paired CCA tissues and normal adjacent tissues. The SKA3 knockdown and overexpression cell line were constructed by small interfering RNA and lentivirus vector transfection. The effect of SKA3 on the proliferation of cholangiocarcinoma under hypoxic conditions was detected by experiments in vitro and in vivo. RNA-seq was used to find out the differentially expressed pathways in cholangiocarcinoma proliferation under hypoxia regulated by SKA3. IP/MS analysis and Western blot assays were used to explore the specific mechanism of SKA3 in regulating the expression of HIF-1a under hypoxia.

**Results:**

SKA3 was up-regulated in NGS, TCGA and GSE107943 databases and was associated with poor prognosis. Functional experiments in vitro and in vivo showed that hypoxia-induced SKA3 promoted cholangiocarcinoma cell proliferation. RNA-sequencing was performed and verified that SKA3 enhanced fatty acid synthesis by up-regulating the expression of key fatty acid synthase, thus promoting cholangiocarcinoma cell proliferation under hypoxic conditions. Further studies indicated that under hypoxic conditions, SKA3 recruited PARP1 to bind to HIF-1a, thus enhancing the poly ADP-ribosylation (PARylation) of HIF-1a. This PARylation enhanced the binding between HIF-1a and USP7, which triggered the deubiquitylation of HIF-1a under hypoxic conditions. Additionally, PARP1 and HIF-1a were upregulated in CCA and promoted CCA cell proliferation. SKA3 promoted CCA cell proliferation and fatty acid synthesis via the PARP1/HIF-1a axis under hypoxic conditions. High SKA3 and HIF-1a expression levels were associated with poor prognosis after surgery.

**Conclusion:**

Hypoxia-induced SKA3 promoted CCA progression by enhancing fatty acid synthesis via the regulation of PARylation-dependent HIF-1a deubiquitylation. Furthermore, increased SKA3 level enhanced chemotherapy-resistance to gemcitabine-based regimen under hypoxic conditions. SKA3 and HIF-1a could be potential oncogenes and significant biomarkers for the analysis of CCA patient prognosis.

**Supplementary Information:**

The online version contains supplementary material available at 10.1186/s13046-023-02842-7.

## Introduction

Cholangiocarcinoma (CCA), which is derived from the epithelium of the bile duct, is a highly malignant tumour with a high mortality rate. CCA can be classified as intrahepatic (ICC), perihilar (pCCA), and distal (dCCA) CCA according to the location of the tumour in the biliary tree [1]. The morbidity rate of CCA has gradually increased over the past 20 years, and CCA has become the second most common primary liver malignancy [2]. Previous studies suggested that surgical resection was the most effective treatment for CCA. Due to the lack of symptoms in the early stage, CCA patients are always diagnosed at advanced stages of disease and have poor prognosis [3, 4]. Therefore, seeking potential therapeutic targets and prognostic predictors for CCA has become increasingly important.

Spindle and kinetochore-associated complex subunit 3 (SKA3) is an essential component of the SKA complex. The SKA complex was reported to function with the KMN network, which includes KNL-1, Mis12, and Ndc80, thus stabilizing the kinetochore–microtubule interaction and promoting mitosis [5, 6]. Decreased SKA3 expression induces mitotic arrest during metaphase by activating the spindle assembly checkpoint and decreasing sister chromatid cohesion [7, 8]. Previous studies have shown that SKA3 is closely related to the occurrence and development of tumours. In hepatocellular carcinoma, SKA3 promotes tumour growth by regulating the phosphorylation of CDK2/P53 [9]. Moreover, SKA3 was also reported to promote proliferation in laryngocarcinoma by activating PLK1-AKT axis-mediated glycolysis [10]. However, the function of SKA3 in CCA and the underlying mechanism remain unknown.

Hypoxia is a common event in solid tumour development that can accelerate tumour growth. When a tumour reaches a volume greater than a few mm^3^, the inner parts of the tumour become characterized by low oxygen levels [10]. The processes by which tumours adapt to hypoxic conditions, including new vessel formation, cancer stem cell (CSC) regulation, glycolysis activation, and fatty acid metabolism, depend on the activation of hypoxia inducible factor (HIF)-1a [11–14]. In the presence of oxygen, HIF-1a can be labelled with ubiquitin and then rapidly degraded via the proteasome [15]. However, the protein expression of HIF-1a is not invariable under hypoxic conditions. Multiple genes, such as GATA3 and CHIP, were reported to regulate the stabilization of HIF-1a under hypoxic conditions [10, 16, 17]. Previous studies have demonstrated that tumour hypoxia and increased HIF-1a expression promote the progression of tumours and tumour resistance to chemotherapy [18]. Therefore, understanding the mechanism underlying HIF-1a regulation under hypoxic conditions is key to developing new strategies for cancer treatment.

PARylation is an important mechanism of protein posttranscriptional modification that widely occurs in living organisms. PARylation is mainly mediated by poly (ADP-ribose) polymerase (PARP), which transfers ADP-ribose to specific amino acid groups of receptor proteins to form poly (ADP-ribose) chains [19]. PARylation can also be mediated by poly (ADP-ribose) hydrolase (PARG) to remove ADP-ribose from receptor proteins. Previous studies have shown that hypoxia induces the activation of PARP1 and then induces the PARylation of HIF-1a, thus stabilizing HIF-1a [10, 20]. However, the specific mechanism underlying the PARP1-induced degradation of the HIF-1a protein remains unclear.

Here, we aimed to elucidate the roles of SKA3 in CCA, as well as the underlying mechanisms, and explore its clinical value. We demonstrated that SKA3 was highly expressed in CCA and associated with poor prognosis. Hypoxia-induced SKA3 expression promoted CCA cell proliferation by enhancing fatty acid synthesis via the PARP1/HIF-1a axis in CCA. High SKA3 level was associated with chemotherapy-resistance to gemcitabine-based regimen under hypoxic conditions. These results suggested that SKA3 and HIF-1a could be potential oncogenes and significant biomarkers for the analysis of CCA patient prognosis.

## Materials and methods

### Human tissue samples and microarray

The tissue microarray was constructed by Outdo Biotech Company (Shanghai, China) from samples that were collected from 110 CCA patients. Tumour and paired para-tumour samples were collected. Para-tumour tissues refers to the normal samples 2cm away from its paired tumour samples. All of the patients underwent surgery at the First Affiliated Hospital of Nanjing Medical University from 2006 to 2017. The expression level of SKA3 was determined by a semiquantitative scoring system. The staining intensity was classified as follows: negative (0), weak (1), moderate (2), or strong (3); the percentage of positive cells in the stained tissues was scored as follows: 0–5% (0), 6–35% (1), 36–70% (2), and > 70% (3). The overall score was calculated by multiplying the staining intensity score and the percentage of positive cells. Two independent pathologists calculated the staining score of each microarray tissue, and the average score was considered the final score. A score of > 6 was considered to indicate upregulated SKA3 expression in tumour tissues; otherwise, SKA3 expression was considered to be downregulated.

The patients were followed up regularly until death or October 25, 2019. The use of clinical samples was approved by the Ethics Committee of The Affiliated Hospital of Nanjing Medical University. Informed consent was obtained in accordance with regional regulations.

### RNA sequencing

Total RNA was extracted from HuCCT1 cells in the negative control and Si-SKA3 groups using TRIzol reagent (Invitrogen, California, USA). The purity and concentration of total RNA were measured by a NanoDrop 2000 nucleic acid protein analyser (Thermo Scientific, MA, USA). RNA-seq analysis was performed by Outdo Biotech Company (Shanghai, China).

### IP coupled with mass spectrometry

Total proteins were extracted from CCA cells, and IP was performed with anti-SKA3 antibodies (ab186003, Abcam) and Protein A/G-agarose beads (Thermo Scientific, MA, USA) as described above. The mass spectrometry analyses were carried out by BGI Tech Solutions Co., Ltd. (BGI Shenzhen, Guangdong, China).

### Statistical analysis

All the statistical analyses were performed with SPSS v26.0 (IBM, SPSS, Chicago, IL, USA) and GraphPad Prism 7 (GraphPad Software, La Jolla, USA). Differences between the two groups were analysed by Student’s t test. The χ^2^ test was used to analyse correlations between SKA3 or HIF-1a expression and clinicopathological variables. The Kaplan‒Meier method and the log-rank test were applied to estimate overall survival (OS) and disease-free survival (DFS). A Cox proportional hazard regression model was used for multivariate analysis. Correlations between SKA3 expression and downstream gene expression were evaluated by Pearson rank correlation analysis. Differences were considered statistically significant when *p < 0.05, **p < 0.01, or ***p < 0.001.

Additional materials and methods are provided in the Supplementary materials and methods.

## Results

### SKA3 was highly expressed in CCA and associated with poor prognosis

**Fig. 1:**
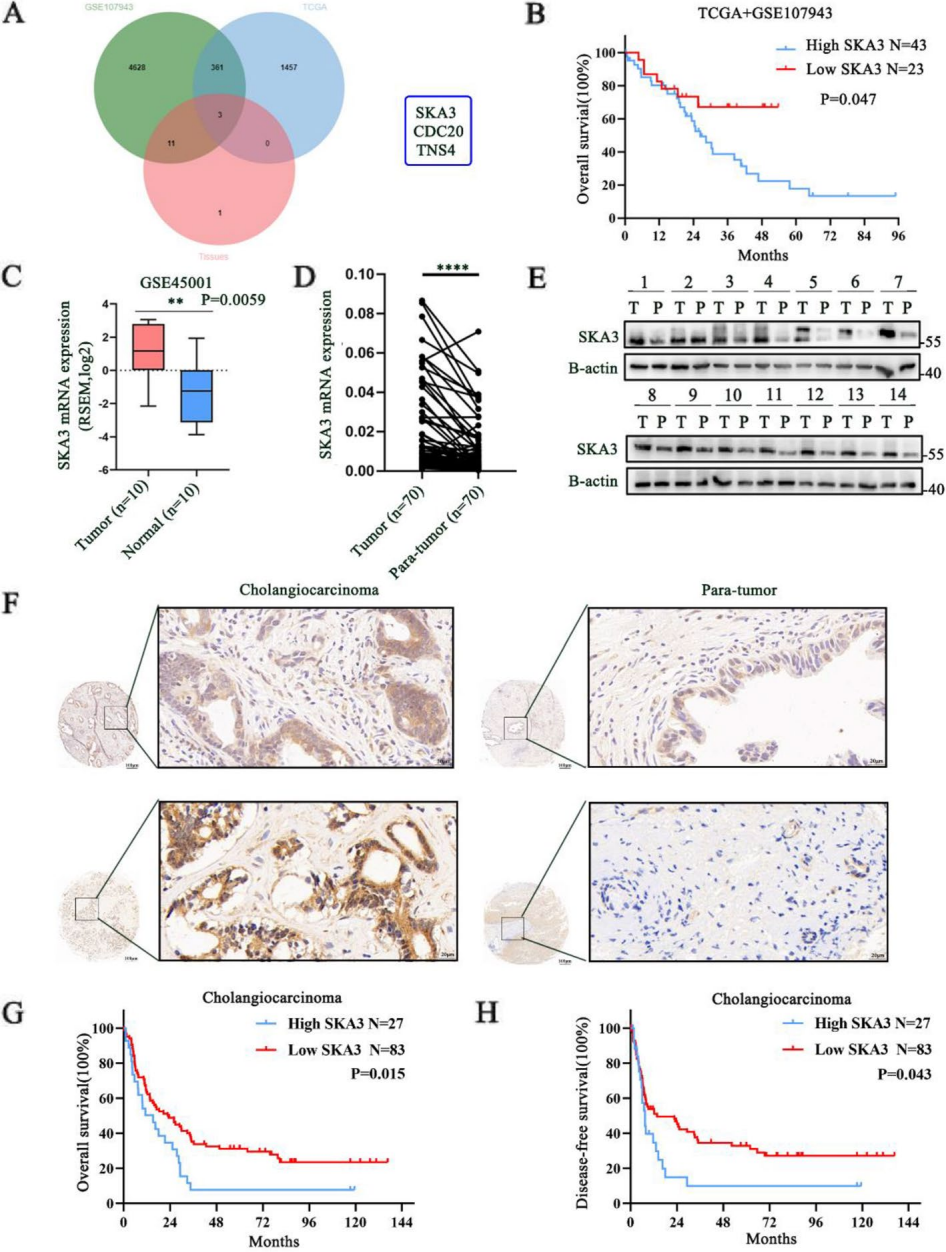
SKA3 was highly expressed in CCA and associated with poor prognosis. **A** Differentially expressed genes searched by TCGA, GSE107943 and Tissues. **B** Prognosis of patients with SKA3 differentially expression in TCGA and GSE107943 datasets. **C** SKA3 was upregulated in GSE45001. **D** RT-qPCR analysis of SKA3 expression in CCA tissues. **E** Western blotting analysis of SKA3 expression in CCA tissues and adjacent normal tissues. **F** IHC of tissue microarrays showed expression of SKA3 was upregulated in CCA tissues (scale bar: 20μm). **G**-**H** Upregulation of SKA3 in CCA was correlated with poor OS and DFS after surgery. *P < 0.05, **P < 0.01, ***P < 0.001

To identify critical genes that contribute to the development of CCA, next-generation sequencing (NGS) was performed with paired CCA tissues and normal adjacent tissues (NATs). Then, we performed a screen to identify upregulated genes in datasets that included patient prognosis information, including The Cancer Genome Atlas (TCGA) and The Gene Expression Omnibus (GEO) dataset (GSE107943). We used criteria (Log_2_FC > 3, *P* < 0.001) to identify upregulated genes, including SKA3, CDC20 and TNS4 in the three datasets (Fig. 1A). Previous studies have shown that CDC20 and TNS4 are closely related to the occurrence and development of CCA [21, 22]. Therefore, we chose SKA3 for further research. TCGA and GSE107943 datasets showed that SKA3 was markedly upregulated in various types of cancers, including CCA (Supplementary 1A-C). To verify the significance of SKA3 in CCA, we established a new dataset, which included the TCGA and GSE107943 datasets, and this new dataset included 66 people and had a rectified batch effect (Supplementary 1D-E). X-tile had been proved to be a new bio-informatics tool for biomarker assessment and survival analysis [23]. In the dataset, we performed survival analysis using the Xtile software found that a high expression level of SKA3 was associated with worse prognosis (Fig. 1B). In addition to this dataset, the upregulation of SKA3 in CCA tissues was also observed in the GSE45001 dataset (Fig. 1C). Then, we explored the mRNA levels of SKA3 in CCA patients at our centre. RT‒qPCR demonstrated that in 70 patients, the mRNA expression of SKA3 was significantly upregulated in CCA tissues compared with paired normal tissues (Fig. 1D). Next, western blotting assays were used to measure the protein expression levels of fourteen pairs of CCA and matched para-normal tissues. The results revealed significantly higher expression of SKA3 in CCA tissues (Fig. 1E), which was consistent with the immunohistochemistry (IHC, Fig. 1F) results. In addition, we measured the mRNA and protein levels of SKA3 in CCA cell lines by RT‒qPCR and western blotting. Compared to the HiBEC normal bile duct cell line, the SKA3 expression level was increased in four CCA cell lines (Supplementary 1F-G).

Subsequently, we analysed the effect of SKA3 on the prognosis of CCA patients and then performed IHC staining on tissue microarrays derived from 110 CCA patients. The association between the SKA3 expression level and the clinicopathological characteristics of the CCA patients is shown in Supplementary Table 1. In addition, the univariate analysis showed that SKA3 expression and lymphatic metastasis were significantly associated with the OS of CCA patients. Furthermore, the multivariate analysis showed that SKA3 expression was an independent predictive factor for postoperative OS (Table 1). Kaplan‒Meier survival curves demonstrated that the high SKA3 expression group had lower overall survival (*P* = 0.015) and disease-free survival (*P* = 0.043) than the low SKA3 expression group (Fig. 1G-H). These results suggested that SKA3 was highly expressed in CCA and associated with poor prognosis.

**Table 1:** Univariate and multivariate analyses of prognostic factors in CCA patients

Variable	Univariate analysis			Multivariate analysis		
	HR	95% CI	*P* value	HR	95% CI	*P* value
Sex	0.643	0.406–1.018	0.060			
Age	0.804	0.519–1.244	0.327			
Tumour size (cm)	1.626	0.984–2.689	0.058			
Differentiation	1.307	0.753–2.269	0.354			
Perineural invasion	1.279	0.766–2.135	0.355			
R0 resection	1.249	0.575–2.711	0.574			
TNM stage	1.378	0.836–2.272	0.209			
Lymphatic metastasis	2.918	1.765–4.821	< 0.001^*^	2.792	1.688–4.619	< 0.001^*^
Vascular Invasion	1.361	0.765–2.423	0.294			
SKA3 Expression	1.795	1.111–2.900	0.017^*^	1.627	1.003–2.641	0.049^*^

^*^P < *0.05*

### SKA3 promoted CCA cell proliferation under hypoxic conditions

**Fig. 2:**
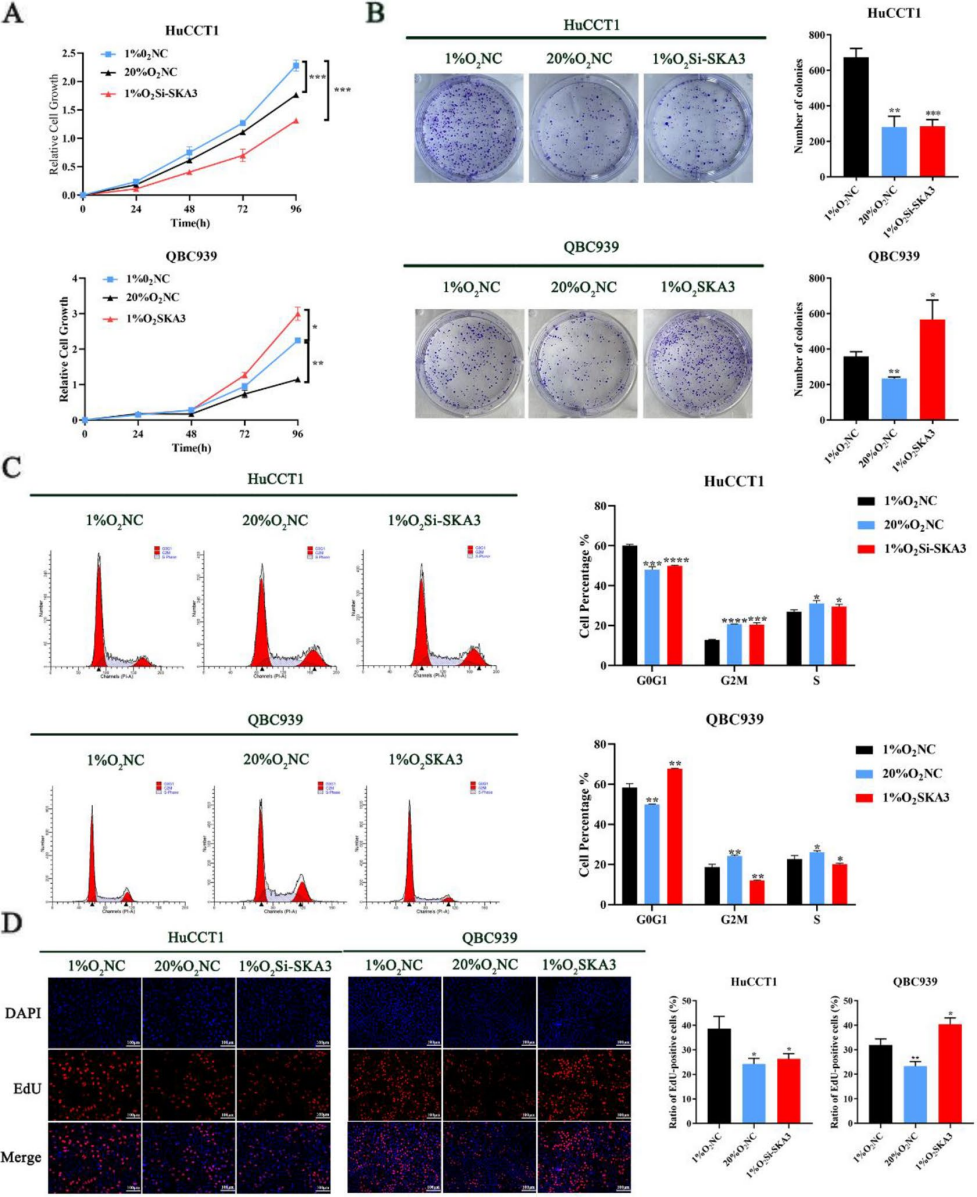
SKA3 promoted CCA cell proliferation under hypoxic conditions. **A** CCK8 assays; **B** clone formation assays; **C** Flow cytometry assays; **D** EdU staining assays indicated that hypoxia-induced SKA3 promoted CCA cell proliferation under hypoxic conditions. *P < 0.05, **P < 0.01, ***P < 0.001

Hypoxia is considered to be an essential feature of the CCA tumour microenvironment (TME). We observed that SKA3 is upregulated under hypoxia in CCA cells (Supplementary 2A). Therefore, we performed further experiments to explore the significance of up-regulated SKA3 expression under hypoxic conditions. According to SKA3 expression in CCA cells, we chose SKA3 high expression HuCCT1 cells to establish a SKA3-knockdown CCA cell line and SKA3 low expression QBC939 cells to establish a SKA3-overexpressing CCA cell line for future functional studies (Supplementary 2B). To investigate the role of SKA3 in the malignant behaviours of CCA under hypoxic conditions, we performed CCK8 assays, clone formation assays, flow cytometry assays and EdU staining assays to further explore the functional changes in SKA3 under hypoxic conditions. First, in the CCK8 assays, under hypoxic conditions, SKA3 knockdown significantly decreased the absorbance at OD450, while SKA3 overexpression increased the absorbance (Fig. 2A). The clone formation assays showed that the number of clones formed by SKA3-knockdown cells was significantly decreased while the number of clones formed by SKA3-overexpressing cells was significantly increased under hypoxic conditions (Fig. 2B). The flow cytometry cell cycle and EdU staining assays demonstrated similar results (Fig. 2C-D). These findings strongly suggested that SKA3 could promote CCA cell proliferation under hypoxic conditions.

### Hypoxia-induced SKA3 promoted CCA progression by enhancing fatty acid synthesis

**Fig. 3:**
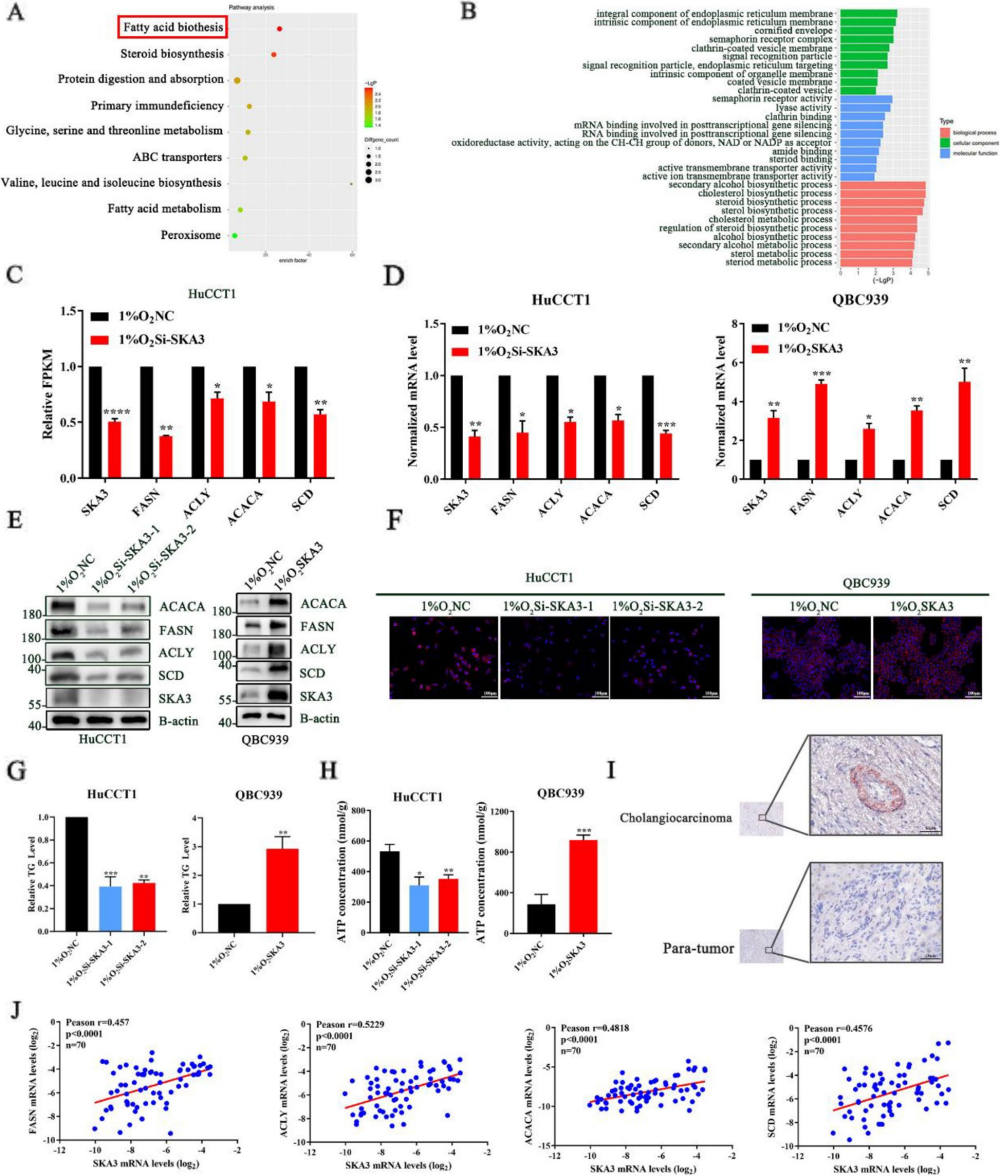
Hypoxia-induced SKA3 promoted CCA progression by enhancing fatty acid synthesis. **A** KEGG pathway analysis of downregulated genes in SKA3-KD HuCCT1 cells. **B** GO analysis of downregulated genes in SKA3-KD HuCCT1 cells. **C** Validation of lipogenic enzyme genes through FPKM normalized from three independent samples. **D** RT-qPCR analysis of mRNA expression of lipogenic enzyme genes FASN, ACLY, ACACA, and SCD in HuCCT1 and QBC939. **E** Protein levels of lipogenic enzymes were determined by western blotting in HuCCT1 and QBC939 cells. **F** Cellular neutral lipids were measured in HuCCT1 and QBC939 cells by double staining with Nile Red and DAPI. **G** Cellular triglycerides were measured in HuCCT1 and QBC939 cells, which normalized by NC group. **H** The ATP concentration in HuCCT1 and QBC939 cells were tested by ATP Assay Kit. **I** Oil red staining was performed in SKA3 highly expressed tumour tissues and paired para-tissues. **J** Scatter plot analysis of correlation between mRNA levels of SKA3 and FASN, ACLY, ACACA, or SCD in 70 CCA tissues. *P < 0.05, **P < 0.01, ***P < 0.001

To reveal the molecular mechanism by which SKA3 is involved in CCA progression under hypoxic conditions, RNA sequencing was carried out to determine the changes in gene expression that occur in HuCCT1-Si-NC and HuCCT1-Si-SKA3 cells after exposure to hypoxia (Supplementary 2C-D). Fatty acid synthesis pathways were identified as the most significantly enriched pathways by Kyoto Encyclopedia of Genes and Genomes (KEGG) analysis (Fig. 3A). GO enrichment showed that secondary alcohol, cholesterol, steroid, and sterol biosynthesis were the main biological processes that were altered (Fig. 3B). Interestingly, the mRNA levels of the key lipogenic enzymes acetyl-CoA carboxylase (ACC), stearoyl-CoA desaturase (SCD), fatty acid synthase (FASN), and ATP-citrate lyase (ACLY) were significantly decreased in HuCCT1-Si-SKA3 cells compared with HuCCT1-Si-NC cells after exposure to hypoxia (Fig. 3C). Then, we performed RT‒qPCR and western blotting assays and further validated that key lipogenic enzymes were decreased in SKA3-knockdown cells under hypoxic conditions but increased in SKA3-overexpressing QBC939 cells (Fig. 3D-E, Supplementary 2E). Next, we performed Nile red staining and found that cellular lipid accumulation was suppressed by SKA3 knockdown but enhanced by SKA3 overexpression group (Fig. 3F). Subsequently, we measured the triglyceride levels of CCA cells and further confirmed that SKA3 knockdown decreased the intracellular levels of triglycerides, but SKA3 overexpression increased these levels in QBC939 cells (Fig. 3G). Then, as demonstrated in Fig. 3H, we measured the ATP concentrations in CCA cells and verified that SKA3 overexpression significantly increased the ATP concentrations in CCA cells, while SKA3 knockdown decreased the ATP concentrations in CCA cells under hypoxic conditions. In summary, SKA3 could reprogram fatty acid metabolism, thus providing energy for CCA cell proliferation under hypoxic conditions. To further confirm our results, we first performed oil red staining assays in CCA tissues and paired para-tumour tissues. As shown in Fig. 3I, we found that the lipid levels in tumour tissues were significantly higher than those in paired para-tumour tissues, which was consistent with the expression of SKA3 shown in Fig. 1E. Subsequently, we performed RT‒qPCR to examine the mRNA levels of ACLY, FASN, ACACA, and SCD in 70 CCA tissue samples. The Pearson correlation analysis showed a significantly positive correlation between SKA3 and FASN (*r* = 0.457, *p* < 0.0001), ACLY (*r* = 0.5229, *p* < 0.0001), ACC (*r* = 0.4818, *p* < 0.0001), and SCD (*r* = 0.4576, *p* < 0.0001) (Fig. 3J). Subsequently, we further investigated whether SKA3 regulates CCA cell proliferation and fatty acid synthesis via regulating all of the four lipogenic enzymes under hypoxic conditions. First, in CCK8 assays, all of the four lipogenic enzymes knockdown reversed the absorbance at OD450 in SKA3-overexpressing cells under hypoxic conditions (Supplementary 3A). The clone formation assays also showed that the increased number of clones formed by SKA3-overexpressing cells could be partly reversed by all of the four lipogenic enzymes knockdown under hypoxic conditions (Supplementary 3B). In addition, Nile red staining, triglyceride level measurements and ATP concentration measurements also showed a similar reversal effect under hypoxic conditions (Supplementary 3C-E). These data suggested that SKA3 enhanced fatty acid synthesis by upregulating all of the four lipogenic enzymes in CCA cells, providing energy for CCA cell proliferation under hypoxic conditions.

### Hypoxia-induced SKA3 interacted with HIF-1a and hindered its degradation in a UPS-dependent manner

**Fig. 4:**
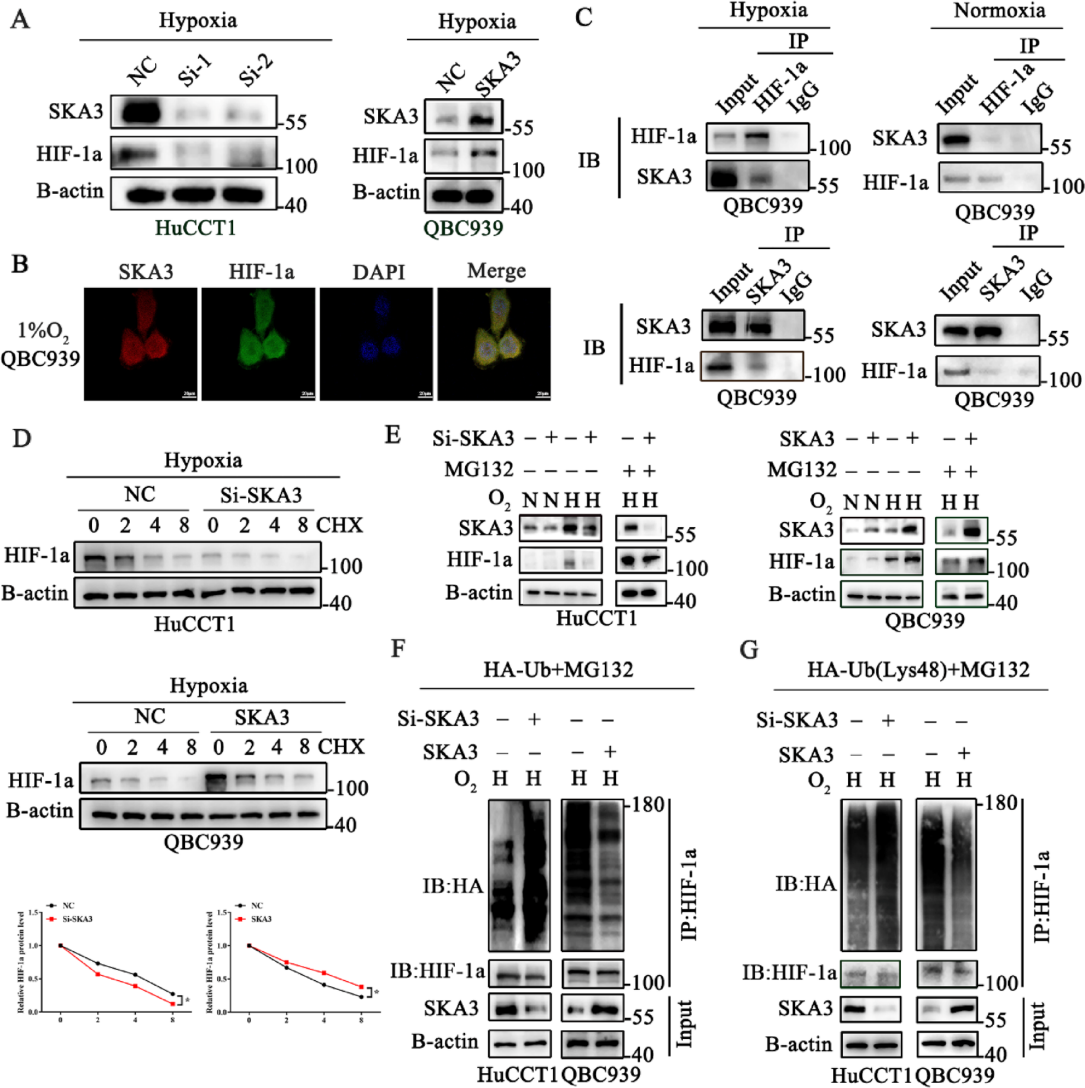
Hypoxia-induced SKA3 interacted with HIF-1a and hindered its degradation in a UPS-dependent manner. **A** Western bolt analysis was used to detect the expression of HIF-1a in SKA3 knockdown and overexpression cells under hypoxic conditions. **B** Immuno-fluorescence technique (IF) verified the co-location between SKA3 and HIF-1a. **C** Co-immunoprecipitation with anti-SKA3, anti-HIF-1a, and anti-IgG was performed to identify the binding between SKA3 and HIF-1a under hypoxic and normoxic conditions. **D** Cells with SKA3 knockdown or overexpression were treated with cyclohexamide (CHX) for indicated time to determine the half-life of HIF-1a protein. **E** Western blotting analysis was used to detect the expression of SKA3 and HIF-1a under normoxic (N) or hypoxic conditions (H) with or without MG132 in SKA3 knockdown and overexpression CCA cells. **F**-**G** Lysates from SKA3 knockdown and SKA3 overexpression CCA cells treated with MG132 and transfected with HA-Ub or HA-Ub lys48 alone under hypoxic conditions, and then the level of ubiquitinated HIF-1a was analysed

HIF-1a is a key molecule in the adaptation of tumour cells to hypoxic environments. However, the specific relationship between SKA3 and HIF-1a in CCA under hypoxic conditions remains unclear. First, we measured the total protein level of HIF-1a in SKA3-knockdown and SKA3-overexpressing cells and found that the protein expression level of HIF-1a was positively correlated with SKA3 (Fig. 4A, Supplementary 4A). Next, we further investigated the mechanism by which SKA3 regulates HIF-1a under hypoxic conditions. We measured the mRNA levels of HIF-1a in SKA3-knockdown and SKA3 overexpression cells under hypoxic conditions and found that the mRNA expression of HIF-1a had no changes (Supplementary 4B). Therefore, we considered that SKA3 regulated HIF-1a expression at the protein level under hypoxic conditions. Subsequently, we performed immunofluorescence (IF) assays and found that SKA3 and HIF-1a were colocalized both in the cytoplasm and nucleus under hypoxic conditions (Fig. 4B). Co-IP analysis was further performed to confirm the interaction between HIF-1a and SKA3. However, no significant interaction between SKA3 and HIF-1a was detected under normoxic conditions (Fig. 4C). Previous studies have shown that in various tumours, including CCA, HIF-1a is degraded in a manner that is dependent on the ubiquitin proteasome system (UPS) [24, 25]. We next explored whether the UPS was involved in SKA3-mediated HIF-1a regulation under hypoxic conditions. First, we found that SKA3 silencing induced HIF-1a degradation, whereas SKA3 overexpression extended the half-life of HIF-1a under hypoxic conditions (Fig. 4D). In addition, HuCCT1-Si-SKA3 cells and QBC939-SKA3-overexpressing cells were treated with the proteasome inhibitor MG132 under hypoxic conditions. After the treatment, the HIF-1a protein level was restored in SKA3-knockdown or SKA3-overexpressing cells under hypoxic conditions. However, the HIF-1a protein level had no changes in SKA3-knockdown or SKA3-overexpressing cells under normoxic conditions (Fig. 4E, Supplementary 4C). Subsequently, we transfected HA-tagged ubiquitin into cells and then administered MG132 to inhibit HIF-1a degradation under hypoxic conditions. When the cells were subjected to immunoprecipitation with anti-HIF-1a antibodies, the results showed that the level of ubiquitinated HIF-1a was increased in SKA3-knockdown cells. Conversely, SKA3 overexpression decreased the level of ubiquitinated HIF-1a (Fig. 4F, Supplementary 4D). To further elucidate the ubiquitinated type of HIF-1a, we transfected Ub-K48-only and MG132 simultaneously into SKA3-knockdown or SKA3-overexpressing CCA cells under hypoxic conditions. Subsequently, we used an anti-HIF-1a antibody to perform protein immunoprecipitation and found that SKA3 knockdown increased K48-linked ubiquitination, while SKA3 overexpression achieved the opposite results (Fig. 4G, Supplementary 4E). These results suggested that hypoxia-induced SKA3 interacted with HIF-1a and then suppressed its degradation in a UPS-dependent manner.

### SKA3 recruited PARP1 to bind to HIF-1a, thus stabilizing HIF-1a in a PAR-dependent manner under hypoxic conditions

**Fig. 5:**
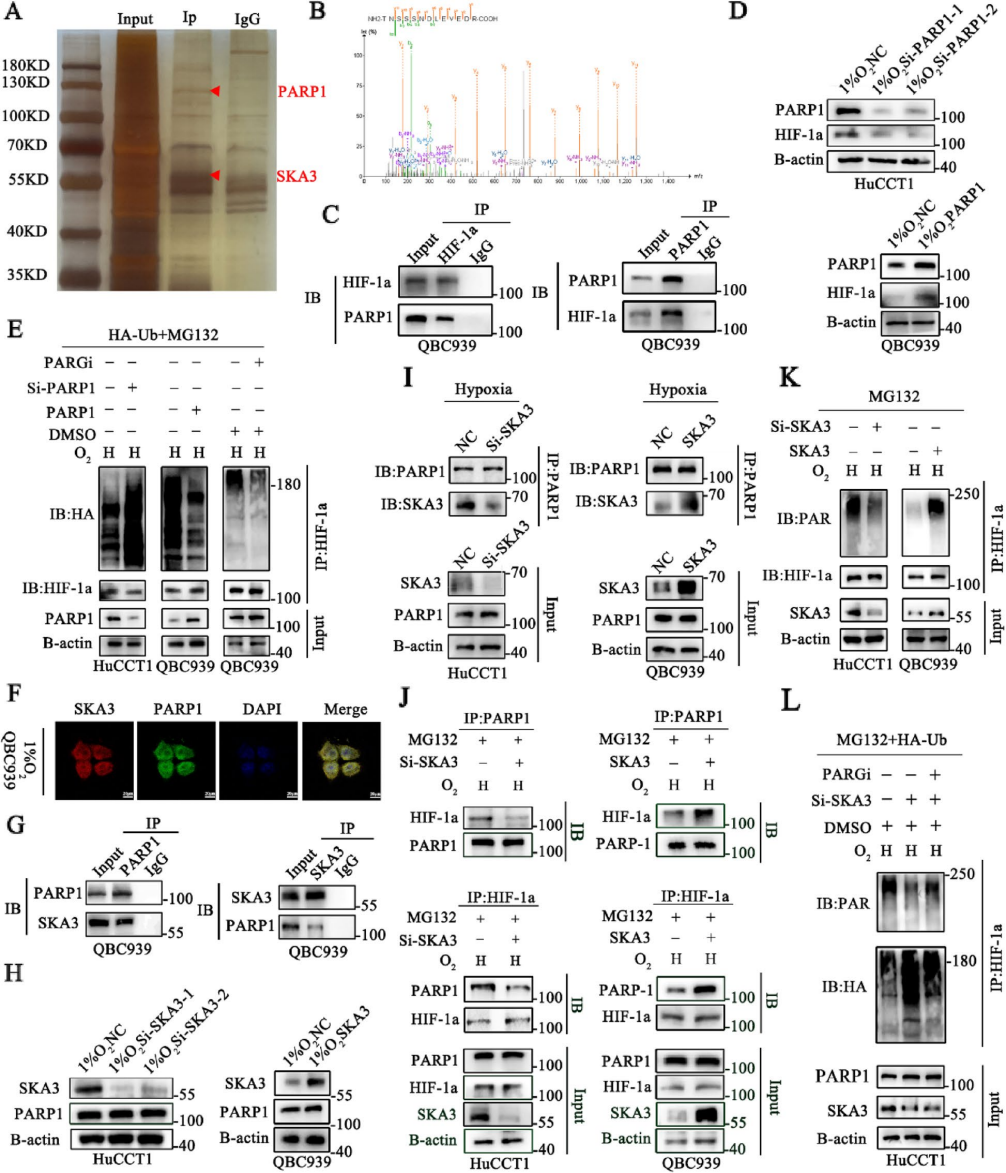
SKA3 recruited PARP1 to bind to HIF-1a, thus stabilizing HIF-1a in a PAR-dependent manner under hypoxic conditions. **A** Sensitive silver staining method was used to seek for differential expressed proteins under hypoxic conditions. **B** IP/MS analysis was used to determine which proteins bind to SKA3, thus identifying PARP1 is an interacting protein under hypoxic conditions. **C** Co-immunoprecipitation with anti-PARP1, anti-HIF-1a or anti-IgG were performed to identify the binding between PARP1 and HIF-1a. **D** Western blotting analysis was used to detect the expression of HIF-1a in PARP1 knockdown and overexpression CCA cells under hypoxic conditions. **E** Lysates from PARP1 knockdown, PARP1 overexpression and PARG inhibitor CCA cells treated with MG132 and transfected with HA-Ub plasmids under hypoxic conditions before collecting were subjected to immunoprecipitation and detected with the indicated antibodies. **F** Immuno-fluorescence technique (IF) verified that SKA3 bound to PARP1 in nucleus and cytoplasm. **G** Co-immunoprecipitation with anti-SKA3, anti-PARP1, and anti-IgG were performed to identify the binding between SKA3 and PARP1. **H** Western bolt analysis was used to detect the expression of PARP1 in SKA3 knockdown and overexpression CCA cells under hypoxic conditions. **I** Western bolt analysis was used to detect the binding ability between PARP1 and SKA3 in SKA3 knockdown and overexpression CCA cells under hypoxic conditions. **J** Western blotting analysis was used to detect the binding ability between PARP1 and HIF-1a in SKA3 knockdown and overexpression CCA cells under hypoxic conditions. **K** Lysates from SKA3 knockdown and overexpression CCA cells treated with MG132 were subjected to immunoprecipitation and detected with the indicated antibodies. **L** The level of ubiquitinated HIF-1a was detected in SKA3 knockdown, PARGi transfected combined with SKA3 knockdown and normal CCA cells under hypoxic conditions

However, SKA3 cannot directly regulate the stabilization of HIF-1a, so we hypothesized that there might be another protein involved in SKA3-mediated HIF-1a stabilization. To obtain deeper insight into the mechanism by which hypoxia-induced SKA3 expression decreased the ubiquitination of HIF-1a, we isolated SKA3-interacting proteins in QBC939 cells under hypoxic conditions and then performed silver staining and IP-coupled MS (IP/MS) to determine which proteins bind to SKA3 (Fig. 5A). Among the SKA3-binding proteins, we identified poly (ADP-ribose) polymerase 1 (PARP1), which was reported to regulate the posttranslational modification of proteins (Fig. 5B). Previous studies reported that in melanoma, PARP1 interacts with the C-terminal domain of HIF-1a and regulates the expression of HIF-1a. These effects are dependent on PARP1 itself or its PARylation activity [10]. However, the specific mechanism underlying PARP1-mediated HIF-1a protein degradation under hypoxic conditions has not been reported. Therefore, we sought to explore whether PARP1 regulates the ubiquitination of HIF-1a in CCA cells under hypoxic conditions. First, we performed co-IP analysis and verified the interaction between PARP1 and HIF-1a in CCA cells under hypoxic conditions (Fig. 5C). Next, we demonstrated that PARP1 knockdown reduced the expression of HIF-1a, while PARP1 overexpression exerted the opposite effect, under hypoxic conditions (Fig. 5D, Supplementary 4F). Furthermore, we transfected a PAR glycohydrolase inhibitor (PARGi) into QBC939 cells and found that hypoxia increased the PARylation activity of PARP1, and the PARylation activity gradually increased with increasing PARGi concentration. The increased PARylation activity was positively associated with the expression of HIF-1a under hypoxic conditions (Supplementary 4G). Finally, we chose 20 µmol/L as the optimal concentration of PARGi under hypoxic conditions for further study. Considering that the UPS is the main mechanism by which proteins are degraded, we subsequently explored the relationship between HIF-1a ubiquitination and PARP1 expression or its PARylation activity. After treatment with MG132, we found that the level of ubiquitinated HIF-1a was increased in PARP1-knockdown HuCCT1 cells and decreased in PARP1-overexpressing or PARGi-treated QBC939 cells under hypoxic conditions (Fig. 5E, Supplementary 5A).

Based on these findings, we investigated the role of SKA3 in the PARP1/HIF-1a complex. As shown in Fig. 5F, immunofluorescence assays verified that SKA3 interacted with PARP1 both in the nucleus and cytoplasm. Co-IP analysis was further performed to confirm the IP/MS results, and the results demonstrated that compared with the IgG group, SKA3 interacted with PARP1 under hypoxic conditions (Fig. 5G). Next, we examined the protein level of PARP1 in SKA3-knockdown HuCCT1 and SKA3-overexpressing QBC939 cells under hypoxic conditions and found that the total expression of PARP1 did not change (Fig. 5H). Then, we suspected that SKA3 stabilized HIF-1a by regulating the PARylation of HIF-1a under hypoxic conditions. First, we investigated the ability of PARP1 and SKA3 to interact in SKA3-knockdown HuCCT1 and SKA3-overexpressing QBC939 cells under hypoxic conditions and found that the interaction between SKA3 and PARP1 was positively associated with the SKA3 expression level, while the total expression of immunoprecipitated PARP1 was not altered (Fig. 5I). Then, we found that after treatment with MG132, the binding of PARP1 to HIF-1a was considerably decreased in SKA3-silenced cells but enhanced in SKA3-overexpressing cells under hypoxic conditions (Fig. 5J). Additionally, we further showed that the amount of PARP1 bound to HIF-1a, which was altered by SKA3 expression, changed the level of PARylated HIF-1a under hypoxic conditions (Fig. 5K, Supplementary 5B). To further explore whether hypoxia-induced SKA3 regulates HIF-1a ubiquitylation in a manner that is dependent on the PARylation of HIF-1a, we transfected PARGi into SKA3-knockdown HuCCT1 cells and found that PARGi significantly reversed the PARylation and ubiquitylation of HIF-1a under hypoxic conditions (Fig. 5L, Supplementary 5C). Based on these findings, we demonstrated that hypoxia-induced SKA3 stabilized HIF-1a in a PAR-dependent manner.

### PARylated HIF-1a triggered its deubiquitylation by USP7 under hypoxic conditions

**Fig. 6:**
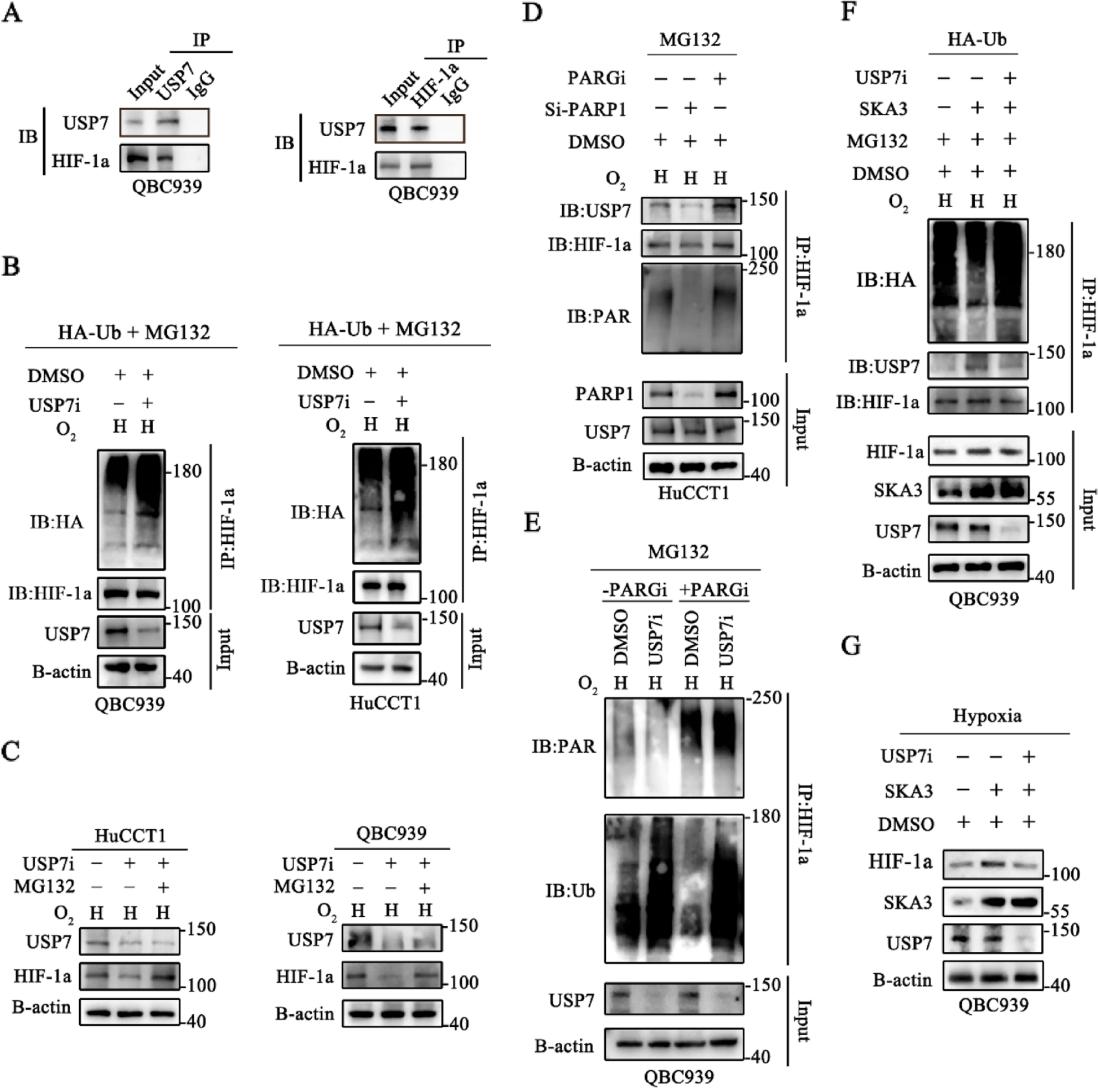
PARylated HIF-1a triggered its deubiquitylation by USP7 under hypoxic conditions. **A** Co-immunoprecipitation with anti-HIF-1a, anti-USP7, and anti-IgG were performed to identify the binding between USP7 and HIF-1a under hypoxic conditions. **B** Lysates from CCA cells treated with MG132 and transfected with HA-Ub and USP7i under hypoxic conditions, and then the level of ubiquitinated HIF-1a was analysed. **C** Inhibit of USP7 promotes degradation of HIF-1a. HuCCT1-USP7i, QBC939-USP7i and their controls were treated with or without MG132. The expression levels of USP7 and HIF-1a were detected. **D** Western blotting analysis was used to detect the binding ability between USP7 and HIF-1a in PARP1 knockdown and PARG inhibit CCA cells under hypoxic conditions. **E** PARylation and ubiquitylation of HIF-1a was detected in four groups under hypoxic conditions, including DMSO, DMSO + USP7i, DMSO + PARGi and DMSO + PARGi + USP7i. **F** Ubiquitylation of HIF-1a under hypoxic conditions was detected in SKA3 overexpression cells with or without USP7i. **G** The total protein level of HIF-1a under hypoxic conditions was detected in SKA3 overexpression cells with or without USP7i

Human ubiquitin-specific protease 7 (USP7), which belongs to the largest USP family of deubiquitinating enzymes (DUBs), was verified to stabilize HIF-1a under hypoxic conditions [26]. First, we performed co-IP assays and verified that USP7 interacted with HIF-1a under hypoxic conditions (Fig. 6A). Furthermore, we found that the transfection of a USP7 inhibitor (USP7i) specifically promoted the ubiquitylation of HIF-1a in HuCCT1 and QBC939 cells under hypoxic conditions (Fig. 6B, Supplementary 5D). Moreover, the depletion of USP7 resulted in a significant decrease in the expression level of HIF-1a in HuCCT1 and QBC939 cell lines, which could be further reversed by MG132 (Fig. 6C, Supplementary 5E).

In a previous study, USP7 was verified to be recruited by PARylated substrates and then trigger their deubiquitylation [27]. However, the roles of USP7 in the PARylation-dependent deubiquitylation of HIF-1a under hypoxic conditions remain unknown. Considering these findings, we explored the amount of USP7 that was bound to HIF-1a in PARP1-knockdown or PARGi-transfected CCA cells and found that the level of PARylated HIF-1a or USP7-bound HIF-1a was significantly decreased in PARP1-knockdown HuCCT1 cells, while the level in PARGi-transfected HuCCT1 cells showed the opposite results (Fig. 6D, Supplementary 5F). We next showed that under hypoxic conditions, PARGi decreased HIF-1a ubiquitylation in the presence of PARGi, which could be reversed by USP7i. Simultaneously, the deubiquitylation of HIF-1a induced by USP7 was significantly enhanced by treatment with PARGi in QBC939 cells under hypoxic conditions (Fig. 6E, Supplementary 5G). To further explore the function of USP7 in the SKA3-mediated deubiquitylation of HIF-1a under hypoxic conditions, we transfected USP7i into SKA3-overexpressing QBC939 cells, and we found that USP7i significantly reversed the SKA3 overexpression-mediated increase in the ubiquitylation of HIF-1a under hypoxic conditions (Fig. 6F, Supplementary 5H). Subsequently, we detected the total protein level of HIF-1a and found that SKA3 overexpression could upregulate the total protein level of HIF-1a, which could be reversed by inhibiting USP7 under hypoxic conditions (Fig. 6G, Supplementary 5I).

Together, these results demonstrated that hypoxia-induced SKA3 could recruit PARP1 to bind to HIF-1a and lead to an increased PARylation of HIF-1a, ultimately stabilizing HIF-1a through its USP7-mediated deubiquitylation.

### HIF-1a promoted CCA cell proliferation and fatty acid synthesis under hypoxic conditions

To further verify the function of HIF-1a in CCA cells, we verified the effect of HIF-1a on CCA proliferation under hypoxic conditions. First, we performed CCK8 assays and found that HIF-1a significantly promoted proliferation in CCA cells under hypoxic conditions (Supplementary 6A). Clone formation assays showed that HIF-1a overexpression significantly increased the numbers of colonies that were formed under hypoxic conditions, while HIF-1a knockdown inhibited clone formation (Supplementary 6B). Flow cytometry cell cycle analyses showed that HIF-1a regulated cell cycle progression through the G2/M and S phase under hypoxic conditions (Supplementary 6C). EdU staining assays further indicated that hypoxia-induced HIF-1a promoted CCA cell proliferation (Supplementary 7). According to a previous study, HIF-1a is closely related to hypoxia-induced fatty acid metabolism [28, 29]. In various types of solid tumours, HIF-1a expression is positively correlated with the expression of FASN, ACLY, ACACA, and SCD [28, 30–33]. Nile red staining showed that HIF-1a knockdown suppressed cellular lipid accumulation, but HIF-1a overexpression enhanced cellular lipid accumulation (Supplementary 8A). Subsequently, the intracellular levels of triglycerides in CCA cells followed the same trends (Supplementary 8B). Then, as demonstrated in Supplementary 8C, we measured the ATP concentrations in CCA cells and verified that HIF-1a knockdown decreased the ATP concentrations in CCA cells, while HIF-1a overexpression significantly increased the ATP concentrations in CCA cells under hypoxic conditions. The western blotting results further verified that HIF-1a could enhance fatty acid synthesis by regulating lipogenic enzymes under hypoxic conditions (Supplementary 8D). All these data demonstrated that HIF-1a promoted cell proliferation and fatty acid synthesis in CCA under hypoxic conditions and was the potential regulatory target of SKA3.

### PARP1 promoted cell proliferation and fatty acid synthesis in CCA under hypoxic conditions

In the TCGA database, we found that PARP1 was highly expressed in various tumours, including CCA (Supplementary 9A-B), and this result was also verified in the GSE107943 database (Supplementary 9C). However, the function of PARP1 in CCA cells under hypoxic conditions is still unclear. In CCK8 assays, we found that PARP1 overexpression significantly promoted, while PARP1 knockdown inhibited, the proliferation of CCA cells under hypoxic conditions (Supplementary 9D). The clone formation assays demonstrated that PARP1 knockdown significantly inhibited the number of colonies that were formed, while PARP1 overexpression enhanced clone formation (Supplementary 9E). Flow cytometry cell cycle analyses and EdU staining assays also showed that hypoxia-induced PARP1 promoted CCA cell proliferation. These results were similar to the above findings (Supplementary 10A-B). Nile red staining and analysis of the triglyceride levels in CCA cells demonstrated that PARP1 could increase the triglyceride levels in CCA cells under hypoxic conditions (Supplementary 11A-B). Moreover, the western blotting results further verified that PARP1 could enhance fatty acid synthesis by regulating lipogenic enzymes under hypoxic conditions (Supplementary 11C). These data showed that PARP1 could promote the proliferation and fatty acid synthesis of CCA cells under hypoxic conditions.

### SKA3 promoted CCA cells proliferation and fatty acid synthesis via the PARP1/HIF-1a axis under hypoxic conditions

**Fig. 7:**
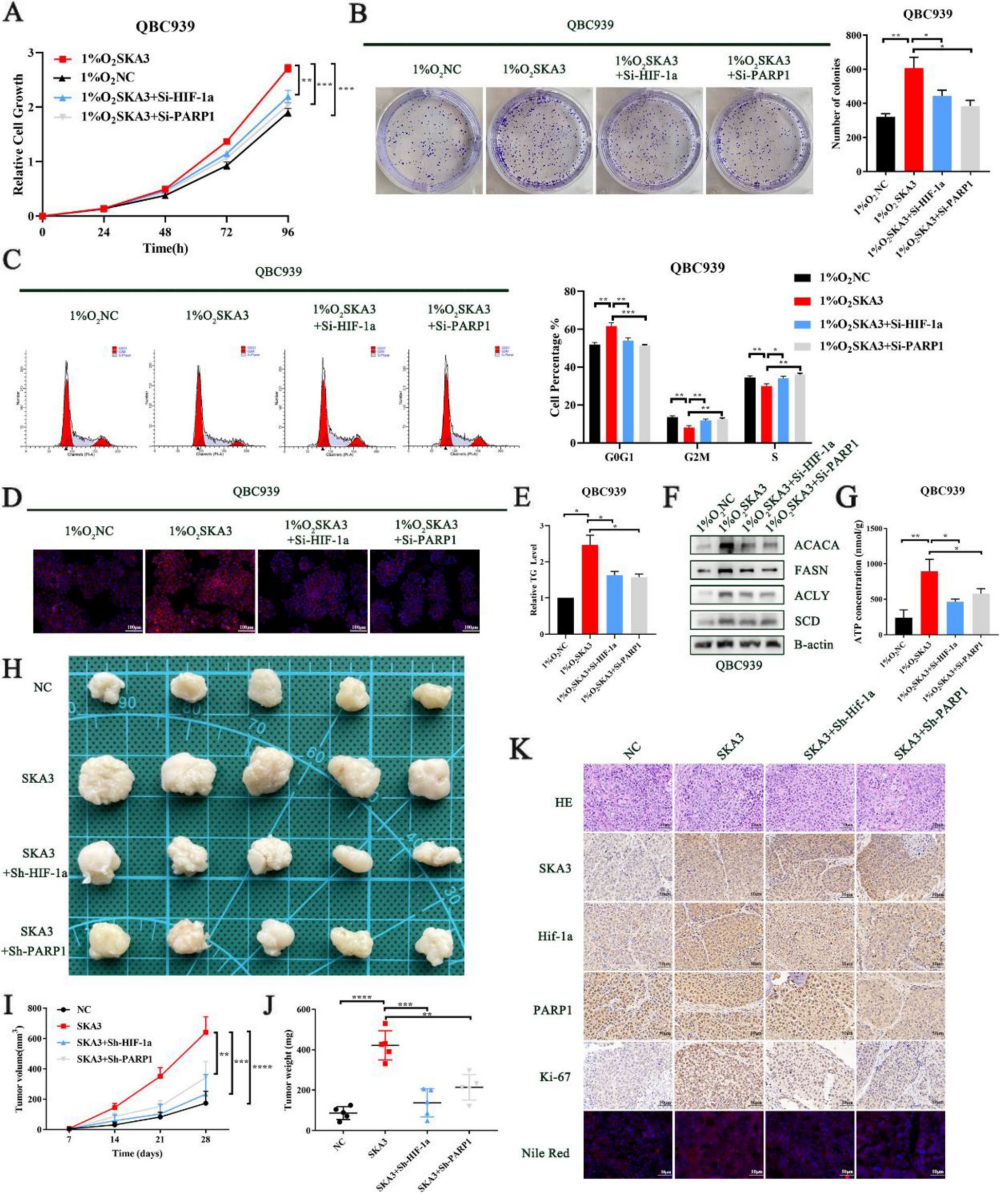
SKA3 promoted CCA cell proliferation and fatty acid synthesis via the PARP1/HIF-1a axis under hypoxic conditions. **A** Knockdown of PARP1 or HIF-1a reversed the proliferation rate of SKA3 overexpression CCA cells under hypoxic conditions. **B** Clone-formation ability of CCA cells was promoted by SKA3 overexpression and rescued by knockdown of PARP1 or HIF-1a under hypoxic conditions. **C** Flow cytometry analysed cell cycle of CCA cells transfected with NC, LV-SKA3, SKA3 overexpression cells transfected with Si-PARP1 and SKA3 overexpression cells transfected with Si-HIF-1a. **D** Cellular neutral lipids were measured in QBC939 cells by double staining with Nile Red and DAPI. **E** Cellular triglycerides were measured in QBC939 cells, normalizing by NC group. **F** Western blotting analysis of fatty acid synthesis markers. **G** The ATP concentration in QBC939 cells were tested by ATP Assay Kit. **H** Xenograft tumours in nude mice generated with QBC939 cells. **I**-**J** Growth of xenografts was promoted in SKA3 overexpression group and rescued in SKA3 overexpression transfected with Si-PARP1 or Si-HIF-1a group. **K** Intensity of Nile red staining and the expression of SKA3, PARP1, HIF-1a and Ki-67 in different groups of xenografts (scale bar: 50μm). *P < 0.05, **P < 0.01, ***P < 0.001

Next, we further investigated whether SKA3 regulates CCA cell proliferation and fatty acid synthesis via the PARP1/HIF-1a axis. As shown in Fig. 7A, PARP1 and HIF-1a knockdown significantly reversed the absorbance at OD450 of SKA3-overexpressing cells under hypoxic conditions. Consistent with the CCK8 assay results, the number of clones that was formed was significantly decreased in SKA3-overexpressing cells with PARP1 and HIF-1a knockdown (Fig. 7B). The cell cycle assays demonstrated similar results (Fig. 7C). In addition, in HuCCT1 cells with SKA3 knockdown, we also found that increased levels of PARP1 and HIF-1a reversed the inhibitory effect of SKA3 knockdown on the proliferation of CCA cells (Supplementary 12A-C). The EdU staining assays also demonstrated that PARP1 and HIF-1a could reverse the impact of SKA3 on the proliferation of CCA cells under hypoxic conditions (Supplementary 12D). Furthermore, Nile red staining demonstrated that PARP1 and HIF-1a knockdown significantly decreased cellular lipid accumulation in SKA3-overexpressing CCA cells (Fig. 7D). The triglyceride level of SKA3-overexpressing CCA cells was also reversed by HIF-1a and PARP1 knockdown (Fig. 7E). Consistent with the functional experiments, western blotting assays showed that the key lipogenic enzymes FASN, ACLY, ACACA and SCD were significantly downregulated in PARP1- and HIF-1a-knockdown CCA cells that overexpressed SKA3 (Fig. 7F, Supplementary 12E). Nile red staining, triglyceride level measurements and western blotting analyses of SKA3-knockdown cells with PARP1 and HIF-1a overexpression showed a similar reversal effect (Supplementary 12F-H). Similar to these results, ATP concentration measurements also demonstrated that SKA3 could regulate the ATP concentration of CCA cells, and this effect could be partly reversed by HIF-1a or PARP1, under hypoxic conditions (Fig. 7G, Supplementary 12I).

Subsequently, in vivo experiments were performed to evaluate the effects of SKA3 on fatty acid synthesis and tumorigenesis. SKA3-overexpressing QBC939 cells, SKA3-overexpressing QBC939 cells with HIF-1a knockdown, and SKA3-overexpressing QBC939 cells with PARP1 knockdown were subcutaneously injected into nude mice. SKA3 overexpression accelerated tumour growth, while these changes were partly reversed by PARP1 and HIF-1a knockdown (Fig. 7H-J). IHC staining of xenografts showed that the expression of Ki-67 was increased in the SKA3 overexpression group. In addition, Nile red staining showed that SKA3 overexpression increased triglyceride levels in tumours, which could be reversed by PARP1 and HIF-1a knockdown (Fig. 7K). Simultaneously, we also found that HIF-1a was expressed at higher levels in SKA3-overexpressing tumours of nude mice than in NC tumours, while PARP1 knockdown reversed this effect. These results revealed that the mechanism by which SKA3 regulated cell proliferation and fatty acid synthesis in CCA was dependent on the PARP1/HIF-1a axis.

### Hypoxia-induced SKA3 protects CCA cells against the cytotoxic effects of gemcitabine

**Fig. 8:**
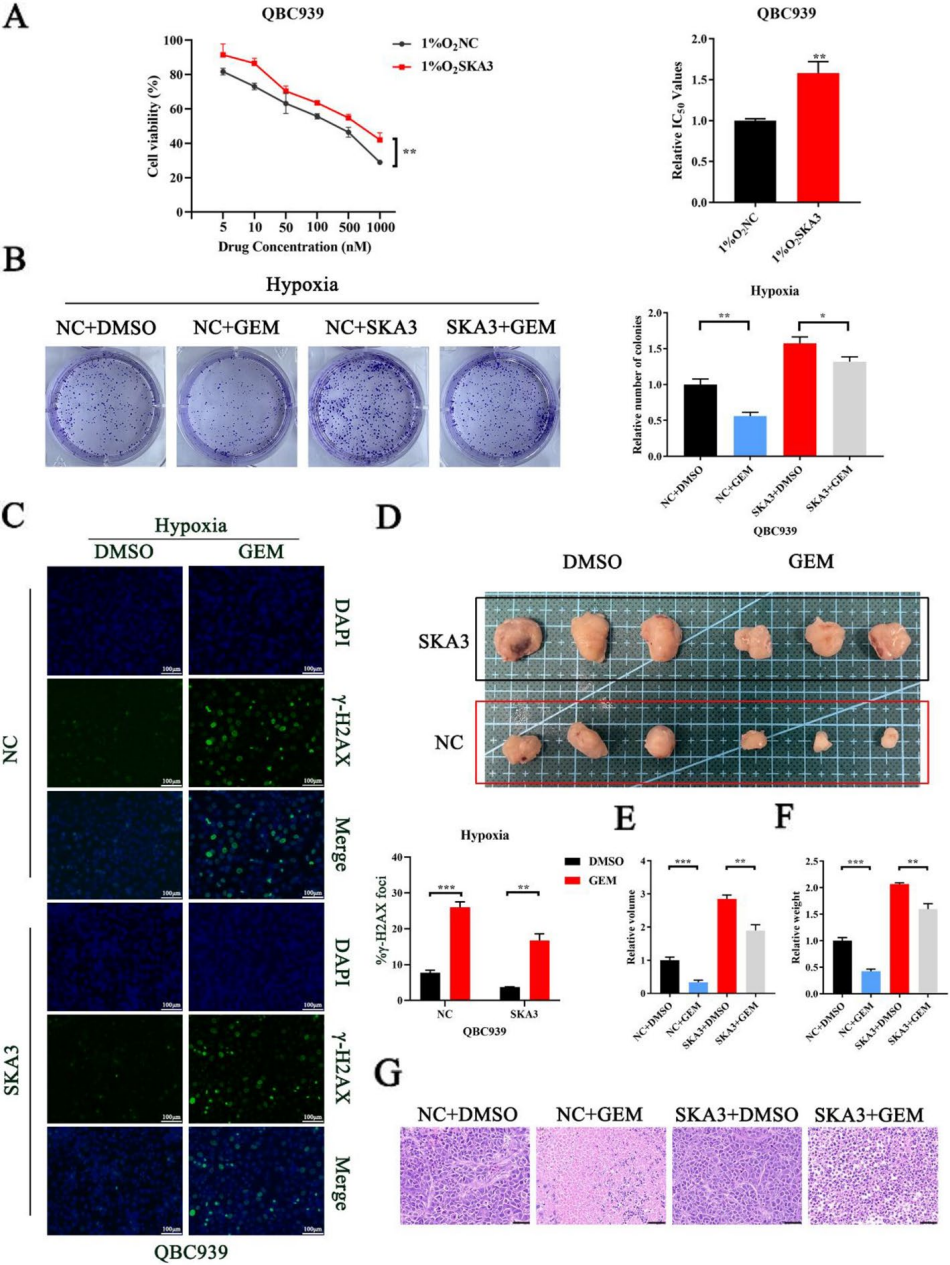
Hypoxia-induced SKA3 protects CCA cells against the cytotoxic effects of gemcitabine. **A** CCA cells with SKA3 overexpression were treated with gemcitabine at different dose under hypoxic conditions, then cell viability was examined after 48 h treatment. **C** CCA cells with SKA3 overexpression group and NC group were treated with gemcitabine at dose of 500 nM/mL under hypoxic conditions, then clone formation assays (**B**) and DNA damage detection assays (**C**) were performed. **D**-**F** The efficacy of gemcitabine in vivo was determined in nude mice injected with SKA3 overexpression QBC939 cells. **G** Representative images of H&E staining in tumour derived from DMSO or gemcitabine-treated nude mice with SKA3 overexpression group and NC group cells inoculation (scale bar: 50μm). *P < 0.05

Currently, gemcitabine-based chemotherapies have been the first-line regimen for advanced biliary tract cancers [34]. Moreover, previous research has proved that lipid metabolism as the metabolic pathway that significantly correlated with poor gemcitabine response [35]. We next determined that whether SKA3 affect the sensitivity of CCA cells to gemcitabine. SKA3 overexpression cells was treated with gemcitabine for 48h with different concentrations of gemcitabine. Subsequently, we detected the viability by CCK8 assay and found that SKA3 overexpression augmented resistance to gemcitabine under hypoxic conditions (Fig. 8A). We observed limited reduction in colony counts when SKA3 overexpression cells were treated with gemcitabine under hypoxic conditions (Fig. 8B). We performed immunofluorescence staining with ant gamma-H2A.X and found that the degree of DNA damage showed less increase in SKA3-overexpressing cells after treated with of gemcitabine under hypoxic conditions compared with NC group (Fig. 8C). The subcutaneous tumour formation experiment in mice showed that gemcitabine obviously decreased the volume and weight of the subcutaneous tumour, while SKA3 overexpression decreased the sensitivity of CCA cells to gemcitabine (Fig. 8D-F). Further examination of the tumour removed from mice showed that gemcitabine enhanced cell necrosis, while SKA3 overexpression weakened its effect (Fig. 8G). The results in vivo and in vitro suggested that SKA3 might be responsible for gemcitabine-resistance in CCA cells.

### SKA3 and HIF-1a were co-expressed in CCA tumours and were reliable combinational biomarkers of CCA prognosis

**Fig. 9:**
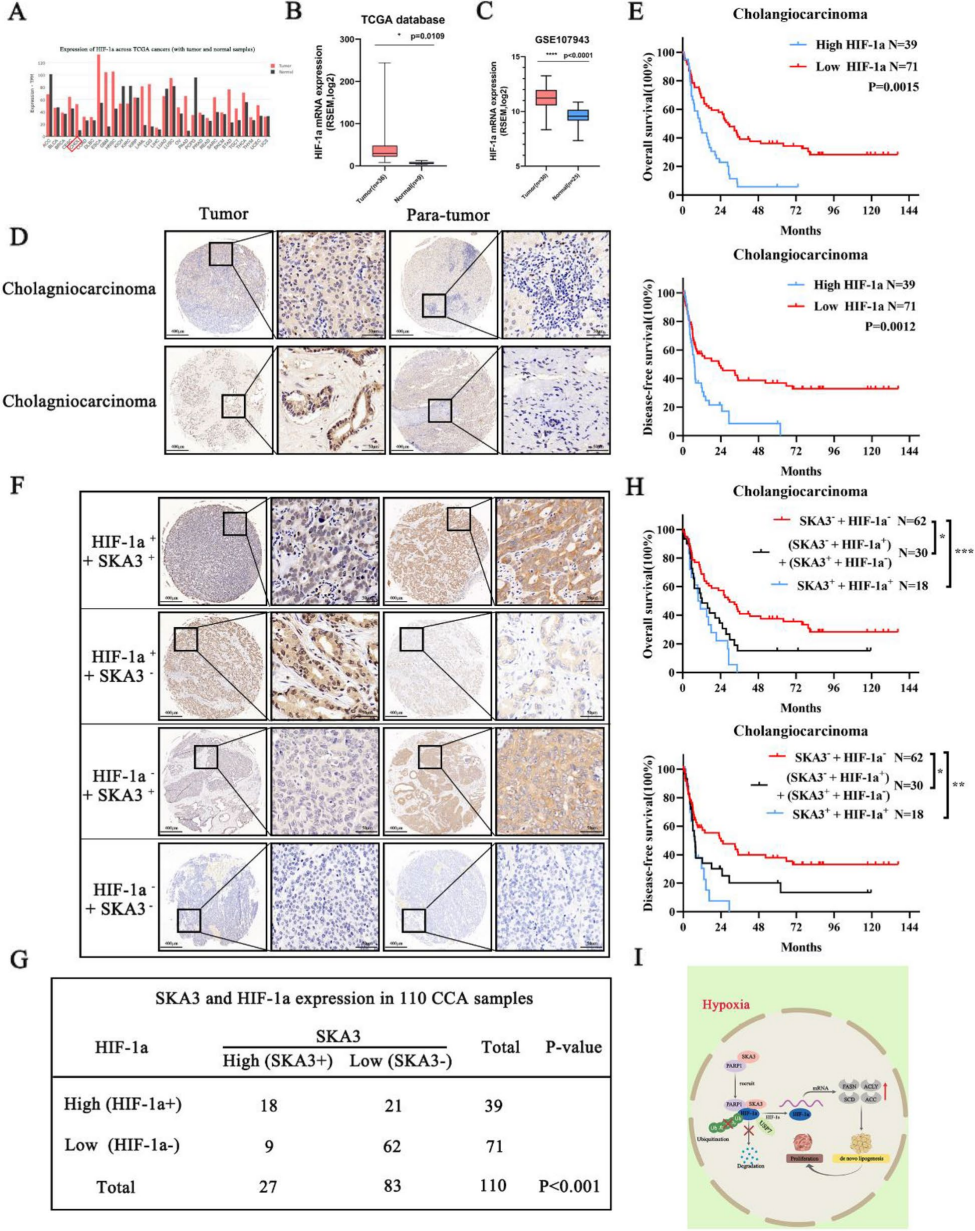
SKA3 and HIF-1a were co-expressed in CCA tumours and were reliable combinational biomarkers of CCA prognosis. **A**-**B** (TCGA) database showed the HIF-1a expression in various of tumours. **C** GSE107943 database demonstrated that HIF-1a was upregulated in cholangiocarcinoma. **D** IHC of tissue microarrays showed expression of HIF-1a was upregulated in CCA tissues (scale bar: 50μm). **E** Upregulation of HIF-1a in CCA was correlated with poor OS and DFS after surgery. **F** IHC assay showed four groups including high HIF-1a and high SKA3 group, high HIF-1a and low SKA3 group, low HIF-1a and high SKA3 group, and high HIF-1a and high SKA3 group (scale bar: 50μm). **G** Correlation of protein expression between SKA3 and HIF-1a. **H** Kaplan–Meier survival curves showed OS and DFS in (SKA3^−^ + HIF-1a^−^) group, (SKA3^−^ + HIF-1a^+^) + (SKA3^+^  + HIF-1a^−^) group, and (SKA3^+^  + HIF-1a.^+^) group. **I** Schematic representation of a model for the major molecular mechanisms of “SKA3-PARP1-HIF-1a” axis-promoted fatty acid synthesis and proliferation in CCA. *P < 0.05

Data from TCGA database demonstrated that HIF-1a was upregulated in various cancer types, including CCA (CHOL) (Fig. 9A-B). In the GSE107943 database, we also found that HIF-1a was significantly highly upregulated in tumour tissues compared with normal tissues (Fig. 9C). Then, we performed immunohistochemical staining on tissue microarrays derived from samples that were collected 110 CCA patients and found that HIF-1a was more highly expressed in CCA tissues than in para-normal tissues (Fig. 9D). The association between the clinicopathological characteristics of CCA patients and HIF-1a protein expression is shown in Supplementary Table 2. Kaplan‒Meier survival curves demonstrated that high expression of HIF-1a was associated with poor prognosis (Fig. 9E). In addition, univariate and multivariate analyses showed that HIF-1a expression and lymphatic metastasis were significantly associated with the OS of CCA patients (Table 2). Additionally, we discovered that the protein expression of HIF-1a was positively correlated with the protein expression of SKA3 (Fig. 9F-G), which further verified the correlation of protein expression between SKA3 and HIF-1a in CCA cells. In addition, compared with patients with low SKA3 levels and low HIF-1a levels (SKA3^−^ + HIF-1a^−^), patients with high SKA3 levels and high HIF-1a levels (SKA3^+^  + HIF-1a^+^) had lower overall survival (*P* = 0.0001) and disease-free survival (*P* = 0.0027). Moreover, patients with high SKA3 or HIF-1a expression ((SKA3^−^ + HIF-1a^+^) + (SKA3^+^  + HIF-1a^−^)) similarly exhibited poor prognosis compared with the (SKA3^−^ + HIF-1a^−^) group (OS: *P* = 0.0261; DFS: *P* = 0.0478) (Fig. 9H). All these data demonstrated that upregulation of HIF-1a was essential for CCA progression and predicted poor survival. SKA3 and HIF-1a might be reliable combinational biomarkers for prognosis as well as therapeutic targets in CCA.

**Table 2:** Univariate and multivariate analyses of prognostic factors in CCA patients

Variable	Univariate analysis			Multivariate analysis		
	HR	95% CI	P value	HR	95% CI	P value
Sex	0.643	0.406–1.018	0.060			
Age	0.804	0.519–1.244	0.327			
Tumour size (cm)	1.626	0.984–2.689	0.058			
Differentiation	1.307	0.753–2.269	0.354			
Perineural invasion	1.279	0.766–2.135	0.355			
R0 resection	1.249	0.575–2.711	0.574			
TNM stage	1.378	0.836–2.272	0.209			
Lymphatic metastasis	2.918	1.765–4.821	< 0.001^*^	2.474	1.477–4.143	< 0.001^*^
Vascular Invasion	1.361	0.765–2.423	0.294			
HIF-1a Expression	2.406	1.527–3.792	< 0.001^*^	2.386	1.474–3.862	< 0.001^*^

^*^P < *0.05*

## Discussion

In this project, we first reported the role of SKA3 in CCA progression and the underlying mechanism. SKA3 was upregulated in CCA tissues and cell lines. Consistent with the expression levels, patients with high SKA3 expression had poorer survival than those with low SKA3 expression. In solid tumours, the inner part of the tumour is characterized by an anoxic environment, which is a key feature of the tumour microenvironment. In this study, we found that SKA3 was up-regulated under hypoxic conditions and our functional experiments further revealed that SKA3 promoted CCA cell proliferation under hypoxic conditions. Based on these findings, SKA3 might be a potential oncogene and crucial biomarker for the prognosis of CCA patients.

Fatty acids are an important energy source for tumour cells, and various cancer cells preferentially use fatty acid synthesis to provide energy for their uncontrolled proliferation [36, 37]. In a variety of human tumours, deregulation of lipogenesis has been considered a carcinogenic event [38–40]. ACLY, FASN, SCD, and ACC are considered key enzymes for fatty acid synthesis and have been reported to promote proliferation in several types of cancers [41–44]. However, the effects of fatty acid synthesis in CCA cells are still unclear. Therefore, a better understanding of fatty acid synthesis in CCA might contribute to the development of new strategies for the treatment of this malignancy. Here, we performed RNA sequencing and found that SKA3 regulated specific groups of genes that are related to FA metabolism. Then, our findings further showed that hypoxia-induced SKA3 was essential for reprogramming FA metabolism in CCA cells by upregulating the key lipogenic enzymes FASN, ACLY, ACC, and SCD. The increasing fatty acid level can provide energy for the uncontrolled proliferation of CCA cells under hypoxic conditions.

The activation of HIF-1a is widely believed to be an essential marker of hypoxic conditions. Previous studies have proven that HIF-1a is positively related to the expression of lipogenic enzymes and promotes fatty acid synthesis in carcinoma [28–33]. In this article, we verified the relationship between HIF-1a and lipogenic enzymes in CCA under hypoxic conditions. Then, we performed functional assays to further confirm the oncogenic function of HIF-1a in CCA cells under hypoxic conditions. We suspected that hypoxia-induced SKA3 might promote fatty acid synthesis by regulating the expression of HIF-1a. We also found that HIF-1a was upregulated in CCA tissues compared with normal tissues, and high HIF-1a expression was associated with poor prognosis and advanced clinicopathological characteristics. The protein expression of HIF-1a was highly positively correlated with SKA3 protein expression in CCA tissues. Patients with high SKA3 levels and high HIF-1a levels always have poor prognoses. However, the specific mechanism by which HIF-1a is regulated by SKA3 under hypoxic conditions remains unclear.

The ubiquitin proteasome system was considered to be the major mechanism underlying the control of HIF-1a stability and activity. Under normoxic conditions, two proline residues (P402 and P564) of HIF-1a are hydroxylated, which triggers its association with Von Hippel‒Lindau (VHL), which is an E3 ubiquitin ligase, leading to the degradation of HIF-1a [45]. In addition to the O_2_/PHD/pVHL ubiquitination pathway, various studies have described oxygen-independent ubiquitination pathways. For instance, heat-shock protein 70 (HSP70) recruit CHIP, which is a new E3 ubiquitin ligase, to bind to HIF-1a, thus regulating the stability of HIF-1a [17]. In addition to this ubiquitination pathway, heat-shock protein 90 (HSP90) and receptor for activated C kinase (RACK1) can competitively bind to HIF-1a, and then, proteasome pathway degradation is initiated [46]. In addition to the ubiquitin proteasome system, many other posttranslational modifications (PTMs), including SUMOylation, phosphorylation, and hydroxylation, have been reported to be modulators of HIF-1a activity [47]. In our studies, we first showed that SKA3 could interact with HIF-1a and regulate the degradation of HIF-1a through the ubiquitin proteasome system under hypoxic conditions. Considering that SKA3 cannot directly regulate the ubiquitylation of HIF-1a, we further explored the potential partner for HIF-1a ubiquitination through IP/MS and identified PARP1. PARP proteins are a family of intracellular enzymes, and PARP1 is the most active member of the family [48]. PARP1 is the member of this family that is most frequently reported to meditate the PARylation of substrates, which is considered a type of reversible PTM and can be blocked by PARGi, thus regulating a wide array of cellular processes, including proteasomal protein degradation, DNA damage and repair, RNA metabolism, and cell death [49, 50]. Additionally, we found that SKA3 did not change total PARP1 expression but interacted with PARP1 and recruited it to bind to HIF-1a under hypoxic conditions, thus altering the level of PARylated HIF-1a. Previous studies have shown that USP7 can regulate the ubiquitylation and degradation of the HIF protein; moreover, USP7 has also been identified as a DUB that reverses PARylation-mediated polyubiquitylation. Given these findings, it can be hypothesized that USP7 is recruited by persistent PARylation to reverse the ubiquitylation of HIF-1a. Our results first demonstrated that USP7 stabilized HIF-1a via a mechanism that depended on the PARylation of HIF-1a, and this process could be regulated by SKA3 under hypoxic conditions. In addition to stabilizing HIF-1a, PARP1 was also reported to recruit HIF-1a to target promoters, thus promoting transcription during hypoxia. The role of SKA3 in this process needs to be verified in future experiments. In addition, in our previous research, we found that SKA3 could not regulate HIF-1a expression in CCA cells under normoxic conditions. This may be due to HIF-1a is heavily degraded under normoxic conditions and has a weak binding capacity to SKA3. Meanwhile, the stability of HIF-1a regulated by SKA3 is dependent on PARylation activity of PARP1, which is significantly activated only under hypoxic conditions. Given these, we considered that there was another possible way that SKA3 promoted CCA progression independent of HIF-1a under normal oxidation conditions, which was the direction of our future research.

## Conclusion

In conclusion, based on these data, we demonstrated that hypoxia-induced SKA3 could recruit PARP1 to bind to HIF-1a and stabilized it through the USP7-mediated deubiquitylation of HIF-1a under hypoxic conditions. The increased level of HIF-1a enhanced triglyceride levels by upregulating key lipogenic enzymes, thus providing an energy source to drive malignant proliferation (Fig. 8I). Furthermore, high expression levels of SKA3 and HIF-1a were associated with poor prognosis after liver surgery. High SKA3 expression promoted the chemotherapy-resistance to gemcitabine treatment, which mean SKA3 could be targeted to improve chemotherapy sensitivity in patients with advanced CCA. These findings indicated that SKA3 might act as a tumour promoter and potential biomarker for CCA. The combination of SKA3 knockdown and HIF-1a inhibition may be a novel strategy for CCA therapy in the future.

### Supplementary Information

**Additional file 1:** **Fig. S1.** (A) SKA3 was upregulated in various types of tumours in the Cancer Genome Atlas (TCGA) database. (B) TCGA database showed that SKA3 was upregulated in CCA.  (C) The Gene Expression Omnibus dataset (GSE107943) indicated that SKA3 was upregulated in CCA tissues. (D-E) Adjust batch effect of TCGA and GSE107943. (F) RT-qPCR and (G) Western blot analysis showed the expression level of SKA3 in CCA cell lines. **Fig. S2.** (A)SKA3 expression was detected under hypoxic and normoxic conditions. (B) SKA3 knockdown and overexpression cells was established in CCA cells. (C) Heatmap and (D) Volcano plot of differential expressed genes, which transfected in NC sequence compared with Si-SKA3 sequence. (E) Statistical and quantitative results of Fig. 3E *p<0.05; **p<0.01; ***p<0.001. **Fig. S3.** (A) CCK8 assays and (B) Clone formation assays showed FASN, ACLY, SCD, ACACA reversed the proliferation of CCA induced by SKA3 under hypoxic conditions. (C) Nile red staining and (D) Cellular triglycerides detection showed FASN, ACLY, SCD and ACACA reversed the fatty acid synthesis of CCA under hypoxic conditions. (E) The ATP concentration in QBC939 cells were tested by ATP Assay Kit. *p<0.05;**p<0.01;***p<0.001. **Fig. S4.** (A) Statistical and quantitative results of Fig. 4A. (B) The mRNA level of HIF-1a was detected in SKA3 knockdown and overexpression cells under hypoxic conditions. (C) Statistical and quantitative results of Fig. 4E. (D) Statistical and quantitative results of Fig. 4F. (E) Statistical and quantitative results of Fig. 4G. (F) Statistical and quantitative results of Fig. 4D. (G) Western blot analysis showed the expression of HIF-1a under hypoxic conditions with the increasing concentration of PARG inhibitor. *p<0.05; **p<0.01; ***p<0.001. **Fig. S5.** Statistical and quantitative results of (A) Fig. 5E, (B) Fig. 5K, (C) Fig. 5L, (D) Fig. 6B, (E) Fig. 6C, (F) Fig. 6D, (G) Fig. 6E, (H) Fig.5F and (G) Fig. 6G. *p<0.05; **p<0.01;***p<0.001. **Fig. S6.** (A) The proliferation of HIF-1a knockdown and overexpression cells under hypoxic conditions was determined by CCK8 assays 0, 24, 48, 72, and 96h . (B) The colony formation number of HIF-1a knockdown and overexpression cells under hypoxic conditions was count. (C) Flow cytometry analyzed cell cycle of CCA cell transfected with Si-HIF-1a or HIF-1a overexpression. *p<0.05; **p<0.01; ***p<0.001. **Fig. S7.** EdU staining assays showed HIF-1a promoted the proliferation of CCA under hypoxic conditions.**Fig. S8.** (A) Cellular neutral lipids were measured in HuCCT1 cells by double staining with Nile Red and DAPI. (B) Cellular triglycerides were measured in HuCCT1 cells, which normalized by NC group. (C) The ATP concentration in HuCCT1 and QBC939 cells were tested by ATP Assay Kit. (D) Western bolt analysis was used to detect the expression of HIF-1a in SKA3 knockdown and overexpression CCA cells under hypoxic conditions.*p<0.05; **p<0.01; ***p<0.001.**Fig. S9.** (A-B) TCGA database showed that PARP1 was oncogenic in various of tumours including CCA. (C) CCK8 assays, and (D) Clone formation assays showed PARP1 promoted the proliferation of CCA under hypoxic conditions.**Fig. S10.** (A) Cell cycle assays and (B) EdU staining assays showed PARP1 enhanced the proliferation of CCA under hypoxic conditions. **Fig. S11.** (A) Nile red staining, (B) Cellular triglycerides detection, and  (C)  Western blot analysis showed PARP1 promoted the fatty acid synthesis of CCA under hypoxic conditions. **Fig. S12.** (A) CCK8 assays, (B) Clone formation assays, (C) Cell cycle analysis, (D) EdU staining assays detected the proliferation of CCA cells. (E) Statistical and quantitative results of Fig. 7F. (F) Nile red staining, (G) Cellular triglycerides detection, and (H) Western blot analysis showed PARP1 or HIF-1a overexpression reversed the increase of fatty acid synthesis in SKA3 Knockdown CCA cells under hypoxic conditions. (I) The ATP concentration in QBC939 cells were tested by ATP Assay Kit.

**Additional file 2.** Supplementary materials and methods.

**Additional file 3:** **Supplementary Table 1.** Association of SKA3 expression with clinicopathologic features of CCA.** Supplementary Table 2.** Association of HIF-1a expression with clinicopathologic features of CCA.

## Data Availability

The experimental data presented in the study are included in the article/ Supplementary Materials, further inquiries can be directed to the corresponding authors upon reasonable request.

## Abbreviations

ACACA: Acetyl-CoA carboxylase
ACLY: ATP-citrate lyase
CCA: Cholangiocarcinoma
Co-IP: Co-immunoprecipitation
DFS: Disease-free survival
EDU: 5-Ethynyl-2’-deoxyuridine
FASN: Fatty acid synthase
GEO: The Gene Expression Omnibus dataset
GEM: Gemcitabine
HIF-1a: Hypoxia inducible factor -1a
HSP90: Heat-shock protein 90
IF: Immunofluorescence
IHC: Immunohistochemistry
IP/MS: Immunoprecipitation coupled mass spectrometry
KEGG: Kyoto Encyclopedia of Genes and Genomes
NGS: Next Generation Sequencing
OS: Overall survival
PARP1: Poly (ADP-ribose) polymerase 1
PARylation: Poly ADP-ribosylation
RACK1: Receptor for activated C kinase
SCD: Stearoyl-CoA desaturase
SKA3: Spindle and kinetochore-associated complex subunit 3
TCGA: The Cancer Genome Atlas
TME: Tumour microenvironment
UPS: Ubiquitin proteasome system
USP7: Human ubiquitin-specific protease 7
VHL: Von Hippel-Lindau
