# Supplementary material for: Hypoxia-induced SKA3 promoted cholangiocarcinoma progression and chemoresistance by enhancing fatty acid synthesis via the regulation of PAR-dependent HIF-1a deubiquitylation
Source: J Exp Clin Cancer Res. 2023 Oct 11;42:265. doi: 10.1186/s13046-023-02842-7 (PMC10565972; doi:10.1186/s13046-023-02842-7)
Supplement: Supplementary file 1 — Additional file 1: Fig. S1. (A) SKA3 was upregulated in various types of tumours in the Cancer Genome Atlas (TCGA) database. (B) TCGA database showed that SKA3 was upregulated in CCA. (C) The Gene Expression Omnibus dataset (GSE107943) indicated that SKA3 was upregulated in CCA tissues. (D-E) Adjust batch effect of TCGA and GSE107943. (F) RT-qPCR and (G) Western blot analysis showed the expression level of SKA3 in CCA cell lines. Fig. S2. (A)SKA3 expression was detected under hypoxic and normoxic conditions. (B) SKA3 knockdown and overexpression cells was established in CCA cells. (C) Heatmap and (D) Volcano plot of differential expressed genes, which transfected in NC sequence compared with Si-SKA3 sequence. (E) Statistical and quantitative results of Fig. 3E *p<0.05; **p<0.01; ***p<0.001. Fig. S3. (A) CCK8 assays and (B) Clone formation assays showed FASN, ACLY, SCD, ACACA reversed the proliferation of CCA induced by SKA3 under hypoxic conditions. (C) Nile red staining and (D) Cellular triglycerides detection showed FASN, ACLY, SCD and ACACA reversed the fatty acid synthesis of CCA under hypoxic conditions. (E) The ATP concentration in QBC939 cells were tested by ATP Assay Kit. *p<0.05;**p<0.01;***p<0.001. Fig. S4. (A) Statistical and quantitative results of Fig. 4A. (B) The mRNA level of HIF-1a was detected in SKA3 knockdown and overexpression cells under hypoxic conditions. (C) Statistical and quantitative results of Fig. 4E. (D) Statistical and quantitative results of Fig. 4F. (E) Statistical and quantitative results of Fig. 4G. (F) Statistical and quantitative results of Fig. 4D. (G) Western blot analysis showed the expression of HIF-1a under hypoxic conditions with the increasing concentration of PARG inhibitor. *p<0.05; **p<0.01; ***p<0.001. Fig. S5. Statistical and quantitative results of (A) Fig. 5E, (B) Fig. 5K, (C) Fig. 5L, (D) Fig. 6B, (E) Fig. 6C, (F) Fig. 6D, (G) Fig. 6E, (H) Fig.5F and (G) Fig. 6G. *p<0.05; **p<0.01;***p<0.001. Fig. S6. (A) The proli [file 13046_2023_2842_MOESM1_ESM.docx]

**Supplementary Figures**

**
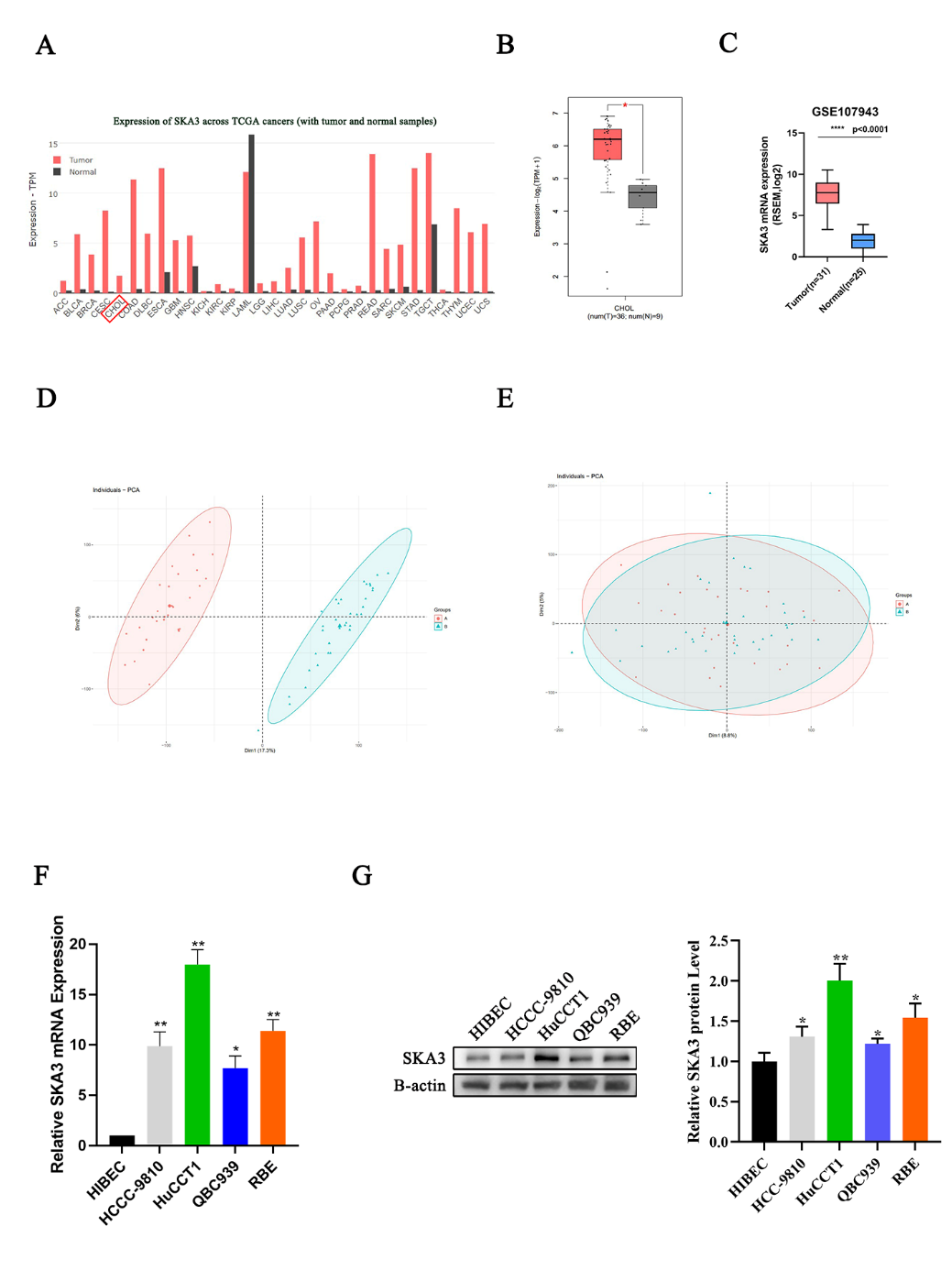
**

**Fig. S1.** (A) SKA3 was upregulated in various types of tumours in the Cancer Genome Atlas (TCGA) database. (B) TCGA database showed that SKA3 was upregulated in CCA. (C) The Gene Expression Omnibus dataset (GSE107943) indicated that SKA3 was upregulated in CCA tissues. (D-E) Adjust batch effect of TCGA and GSE107943. (F) RT-qPCR and (G) Western blot analysis showed the expression level of SKA3 in CCA cell lines.


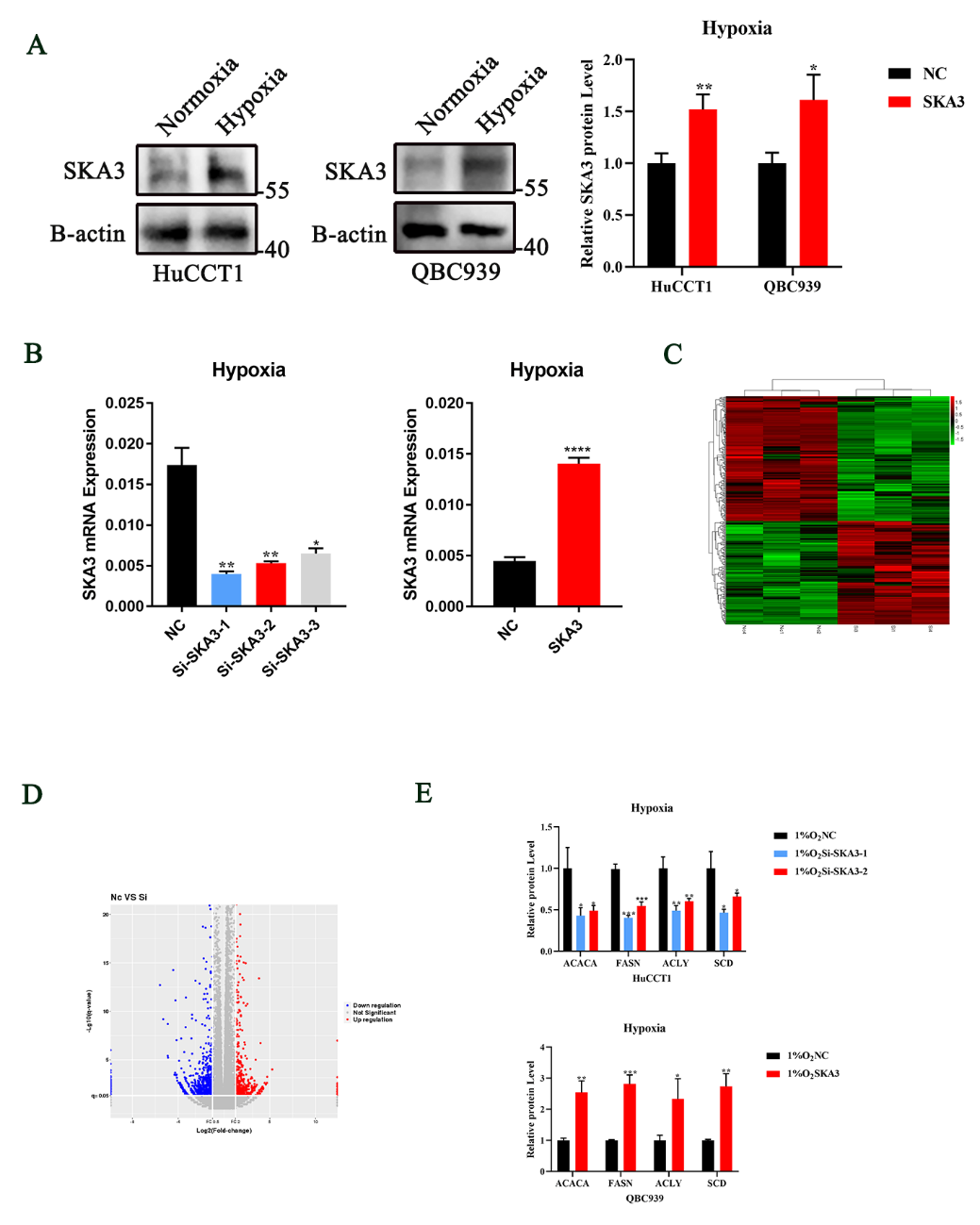


**Fig. S2.** (A)SKA3 expression was detected under hypoxic and normoxic conditions. (B) SKA3 knockdown and overexpression cells was established in CCA cells. (C) Heatmap and (D) Volcano plot of differential expressed genes, which transfected in NC sequence compared with Si-SKA3 sequence. (E) Statistical and quantitative results of Figure 3E *p<0.05; **p<0.01; ***p<0.001.


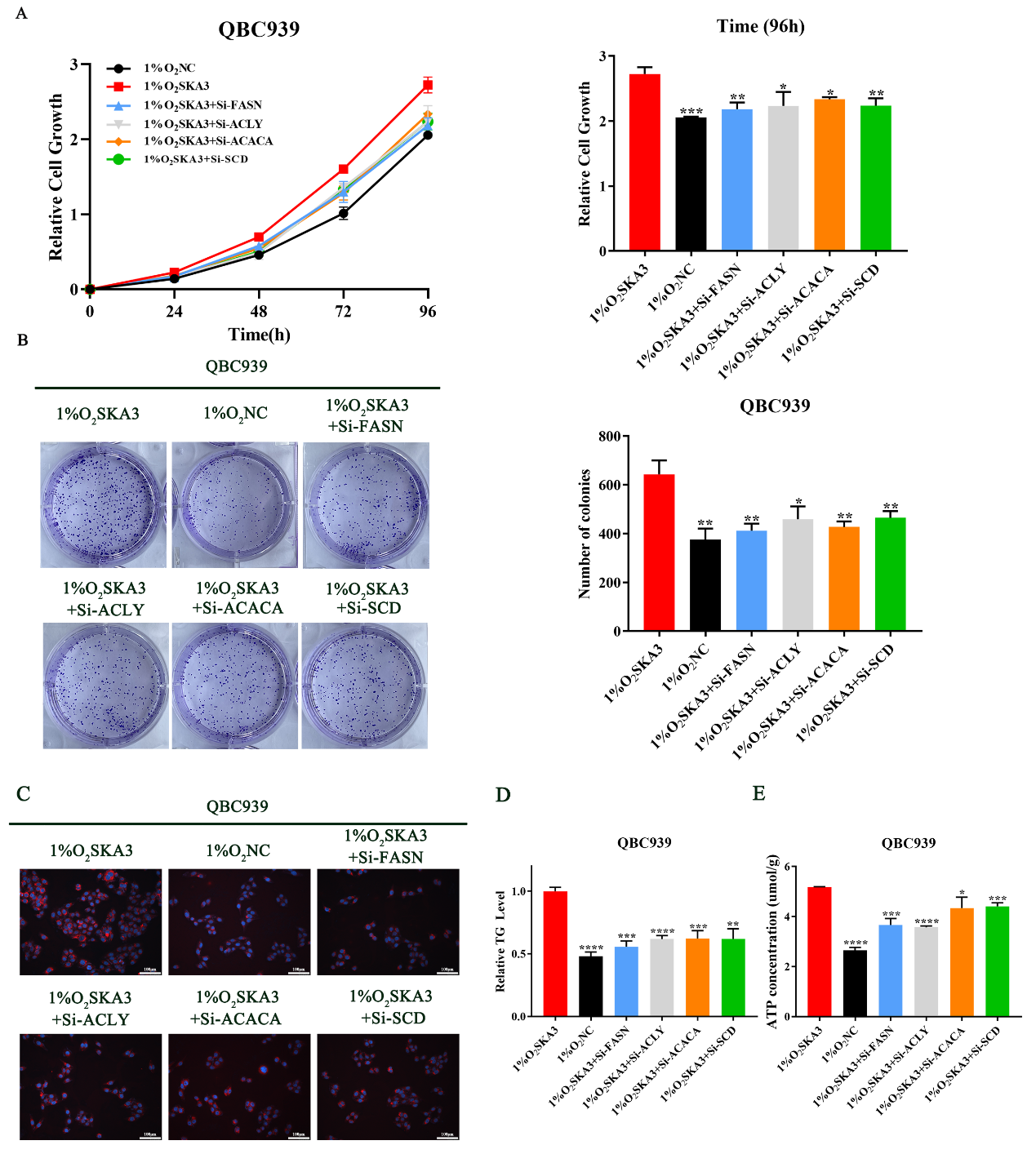


**Fig. S3.** (A) CCK8 assays and (B) Clone formation assays showed FASN, ACLY, SCD, ACACA reversed the proliferation of CCA induced by SKA3 under hypoxic conditions. (C) Nile red staining and (D) Cellular triglycerides detection showed FASN, ACLY, SCD and ACACA reversed the fatty acid synthesis of CCA under hypoxic conditions. (E) The ATP concentration in QBC939 cells were tested by ATP Assay Kit. *p<0.05; **p<0.01;***p<0.001.
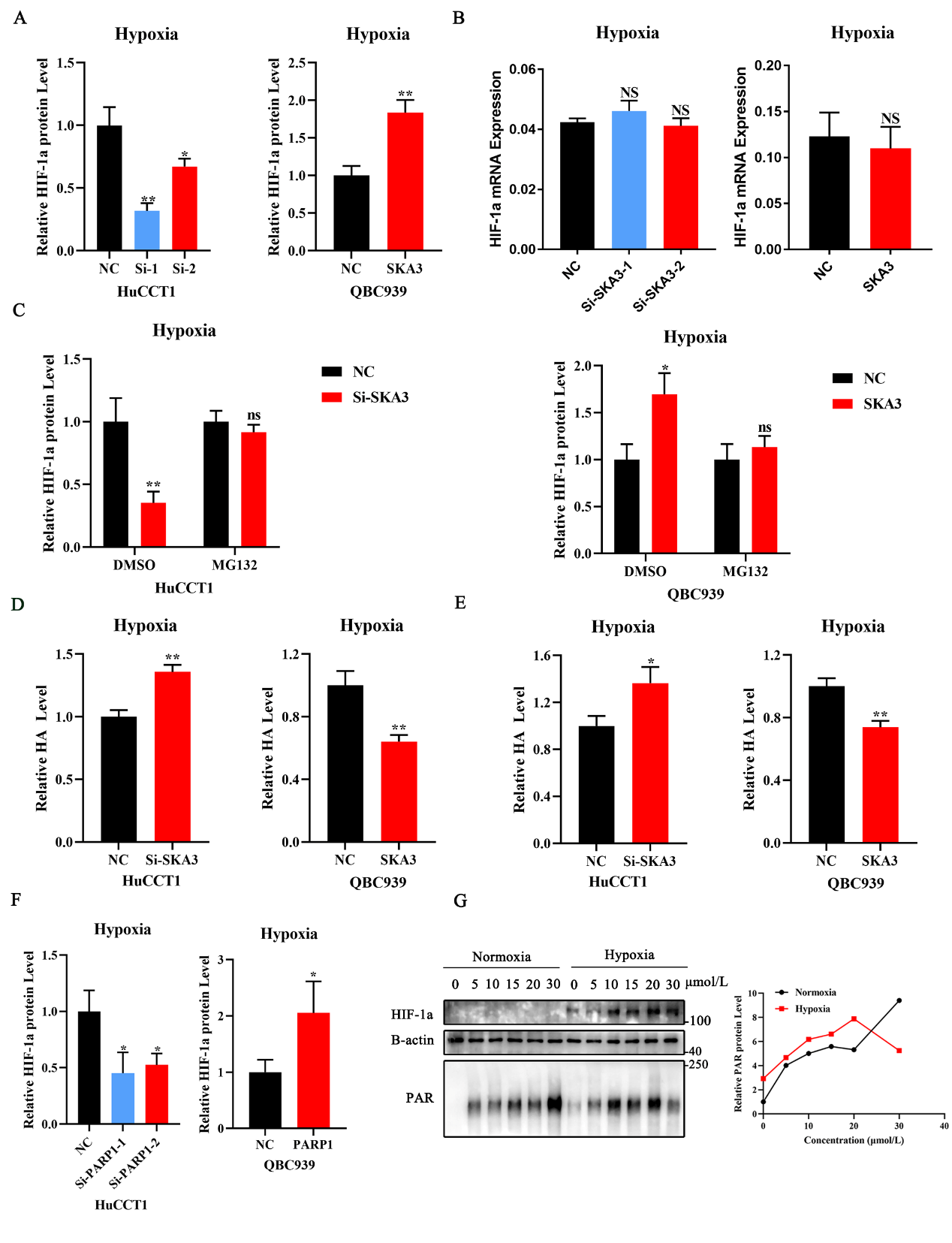


**Fig. S4.** (A) Statistical and quantitative results of Figure 4A. (B) The mRNA level of HIF-1a was detected in SKA3 knockdown and overexpression cells under hypoxic conditions. (C) Statistical and quantitative results of Figure 4E. (D) Statistical and quantitative results of Figure 4F. (E) Statistical and quantitative results of Figure 4G. (F) Statistical and quantitative results of Figure 4D. (G) Western blot analysis showed the expression of HIF-1a under hypoxic conditions with the increasing concentration of PARG inhibitor. *p<0.05; **p<0.01; ***p<0.001.


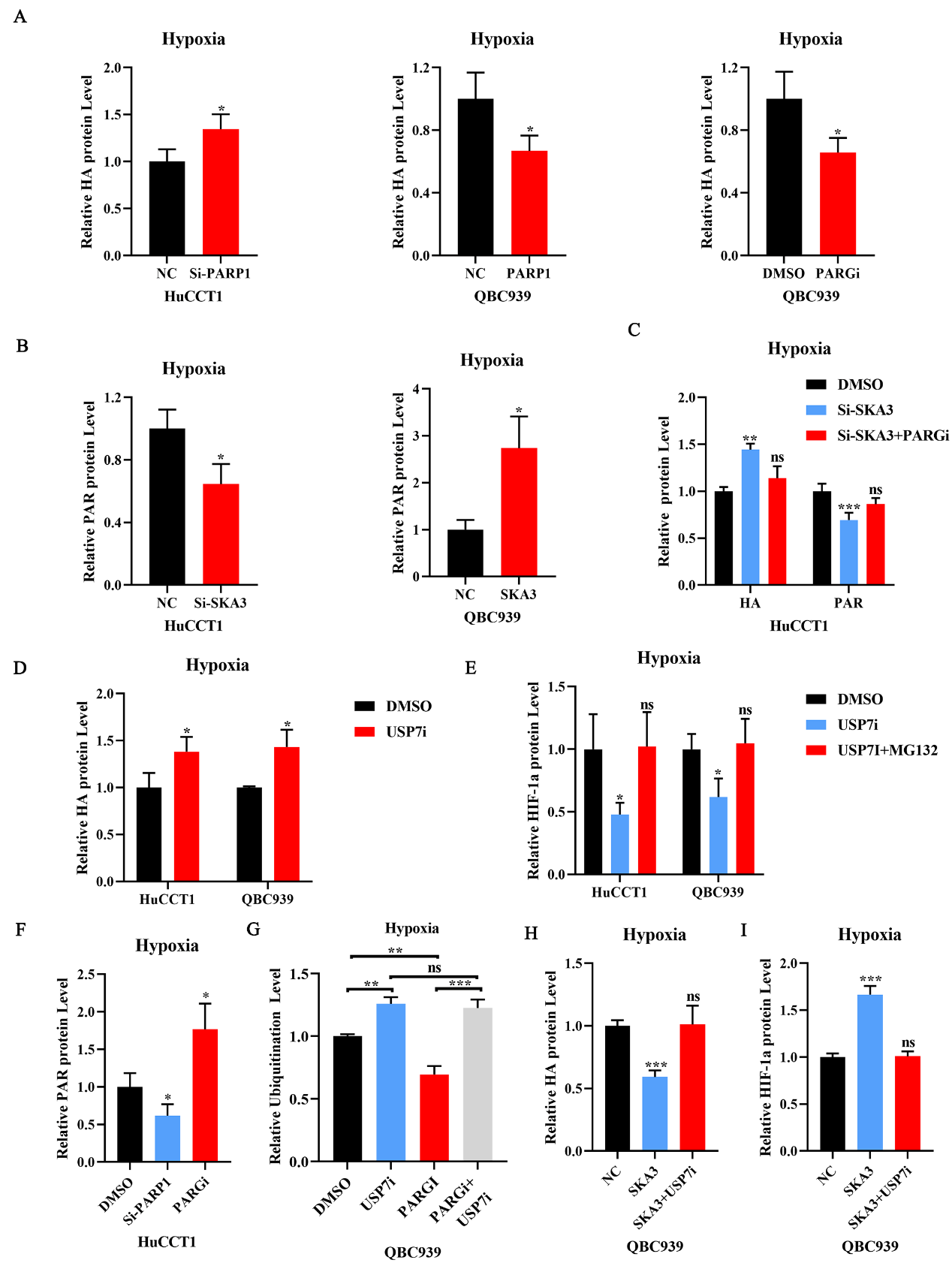


**Fig. S5.** Statistical and quantitative results of (A) Figure 5E, (B) Figure 5K, (C) Figure 5L, (D) Figure 6B, (E) Figure 6C, (F) Figure 6D, (G) Figure 6E, (H) Figure 5F and (G) Figure 6G. *p<0.05; **p<0.01; ***p<0.001.


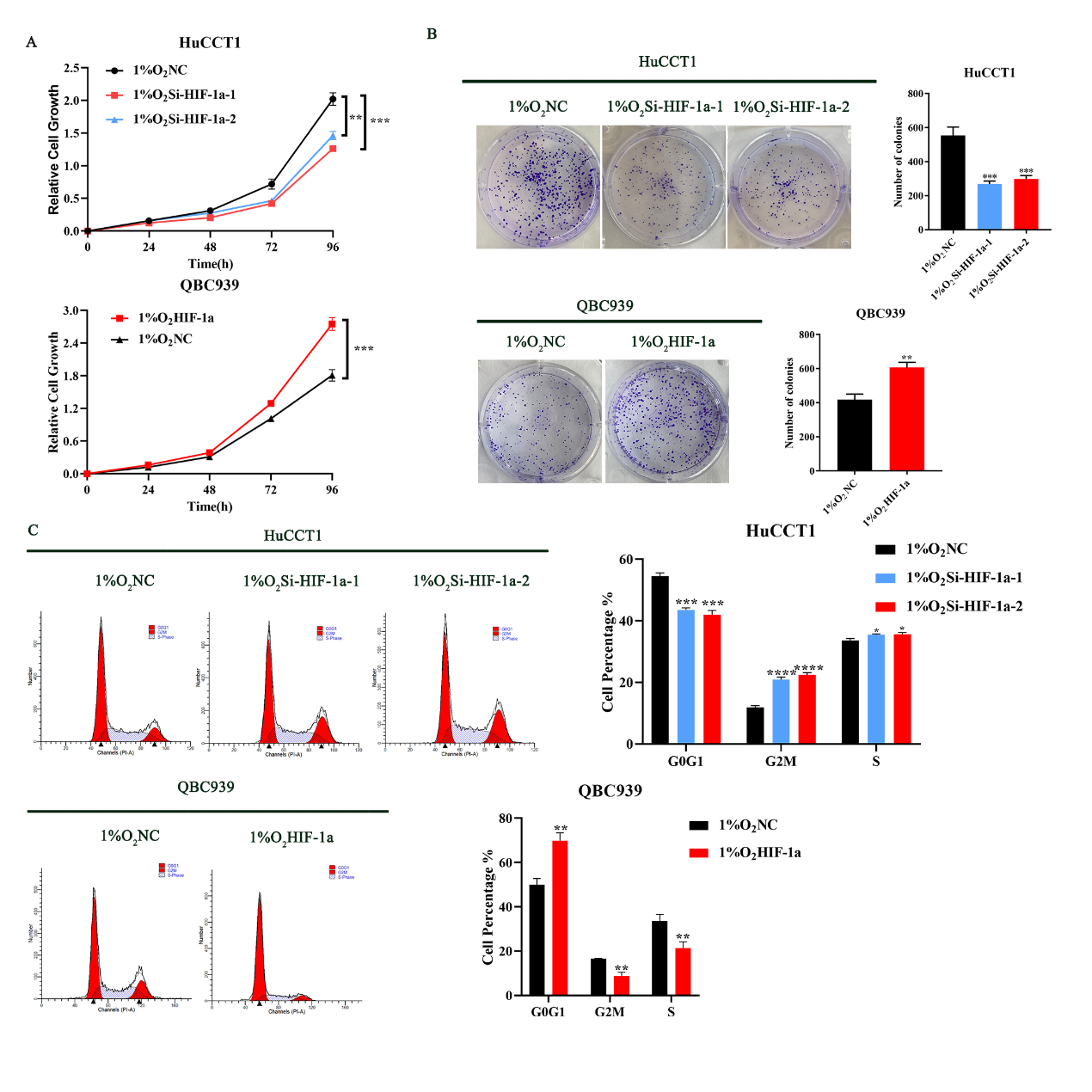


**Fig. S6.** (A) The proliferation of HIF-1a knockdown and overexpression cells under hypoxic conditions was determined by CCK8 assays 0, 24, 48, 72, and 96h . (B) The colony formation number of HIF-1a knockdown and overexpression cells under hypoxic conditions was count. (C) Flow cytometry analyzed cell cycle of CCA cell transfected with Si-HIF-1a or HIF-1a overexpression. *p<0.05; **p<0.01; ***p<0.001.


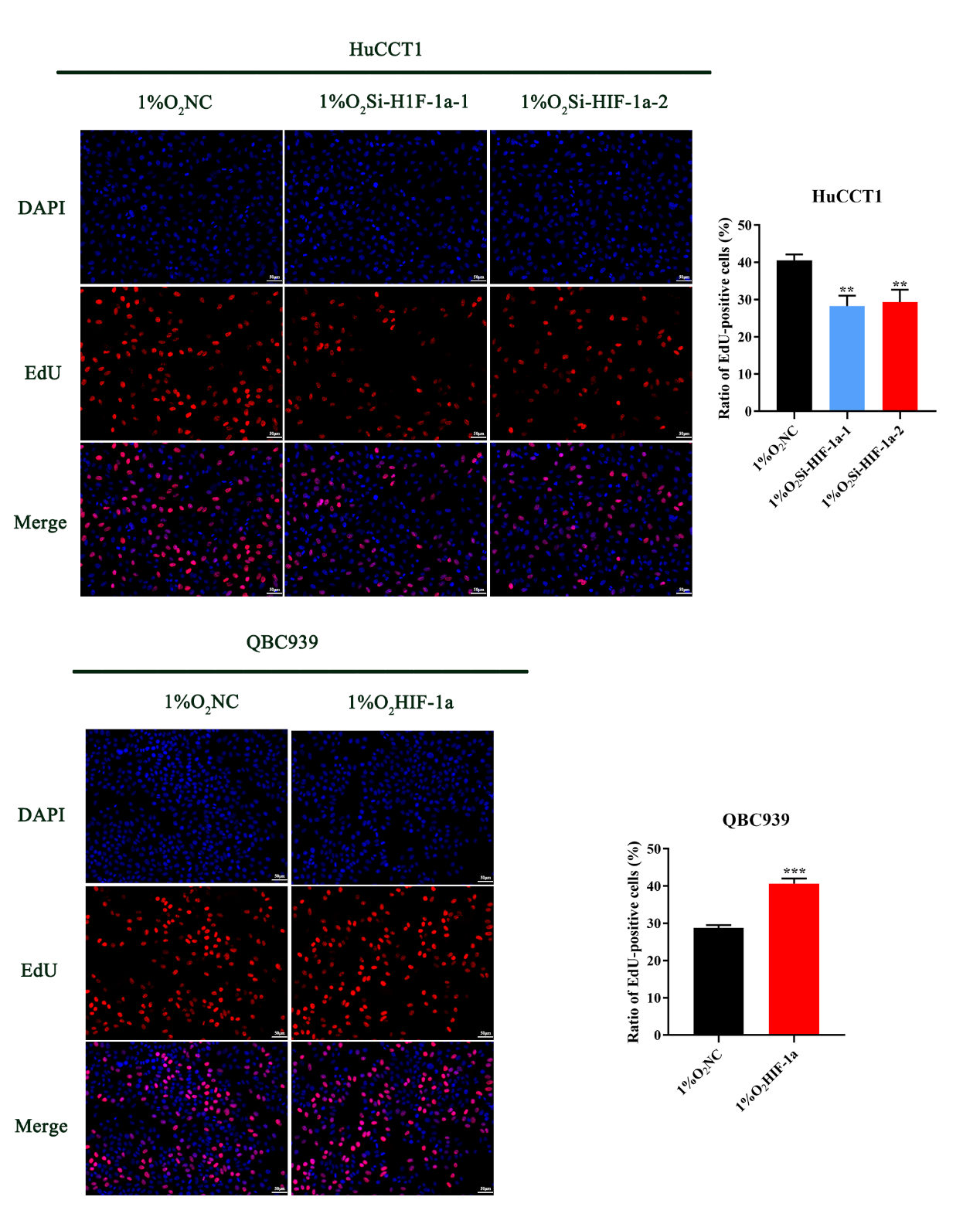


**Fig. S7.** EdU staining assays showed HIF-1a promoted the proliferation of CCA under hypoxic conditions.


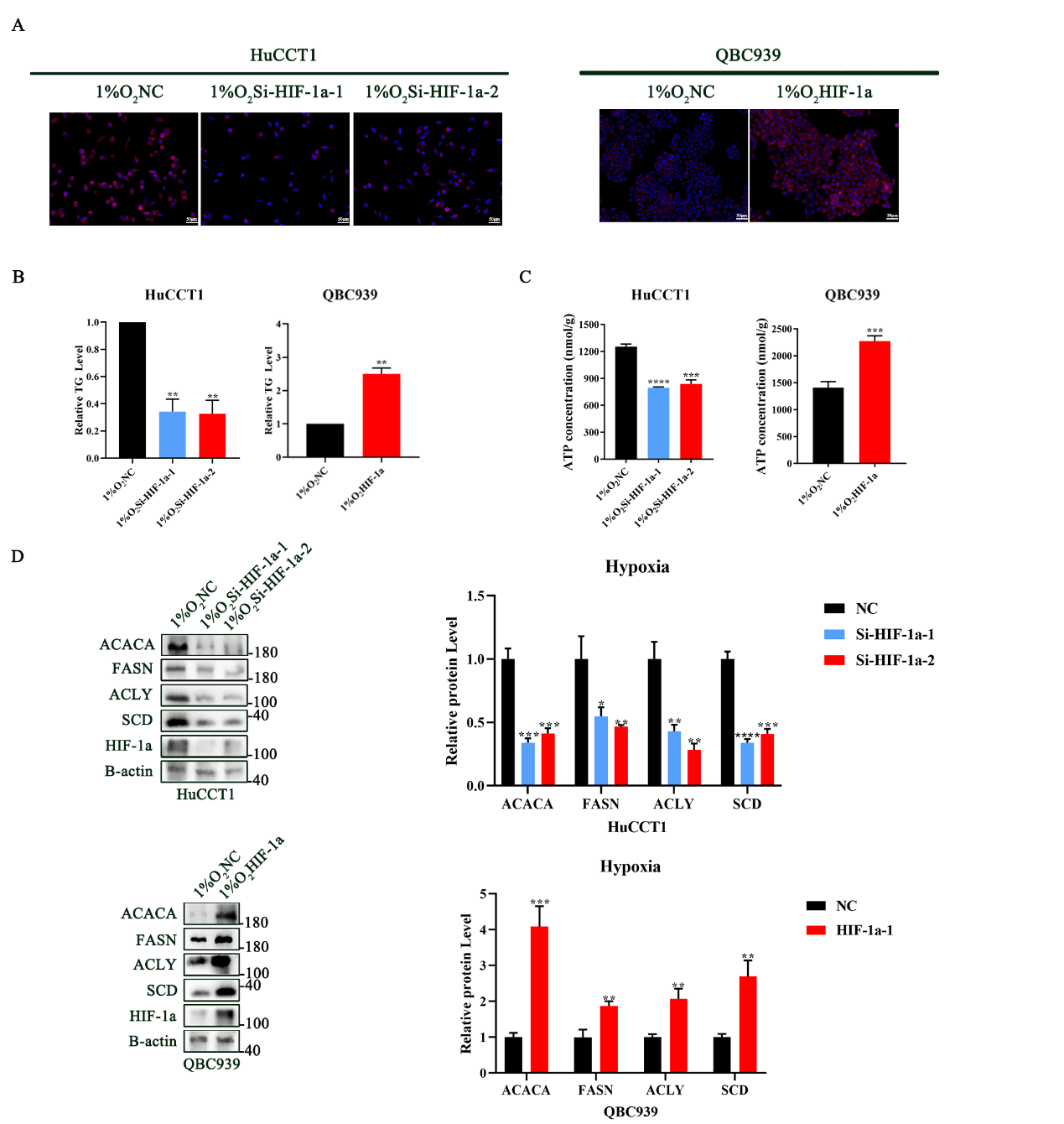


**Fig. S8.** (A) Cellular neutral lipids were measured in HuCCT1 cells by double staining with Nile Red and DAPI. (B) Cellular triglycerides were measured in HuCCT1 cells, which normalized by NC group. (C) The ATP concentration in HuCCT1 and QBC939 cells were tested by ATP Assay Kit. (D) Western bolt analysis was used to detect the expression of HIF-1a in SKA3 knockdown and overexpression CCA cells under hypoxic conditions. *p<0.05; **p<0.01; ***p<0.001.


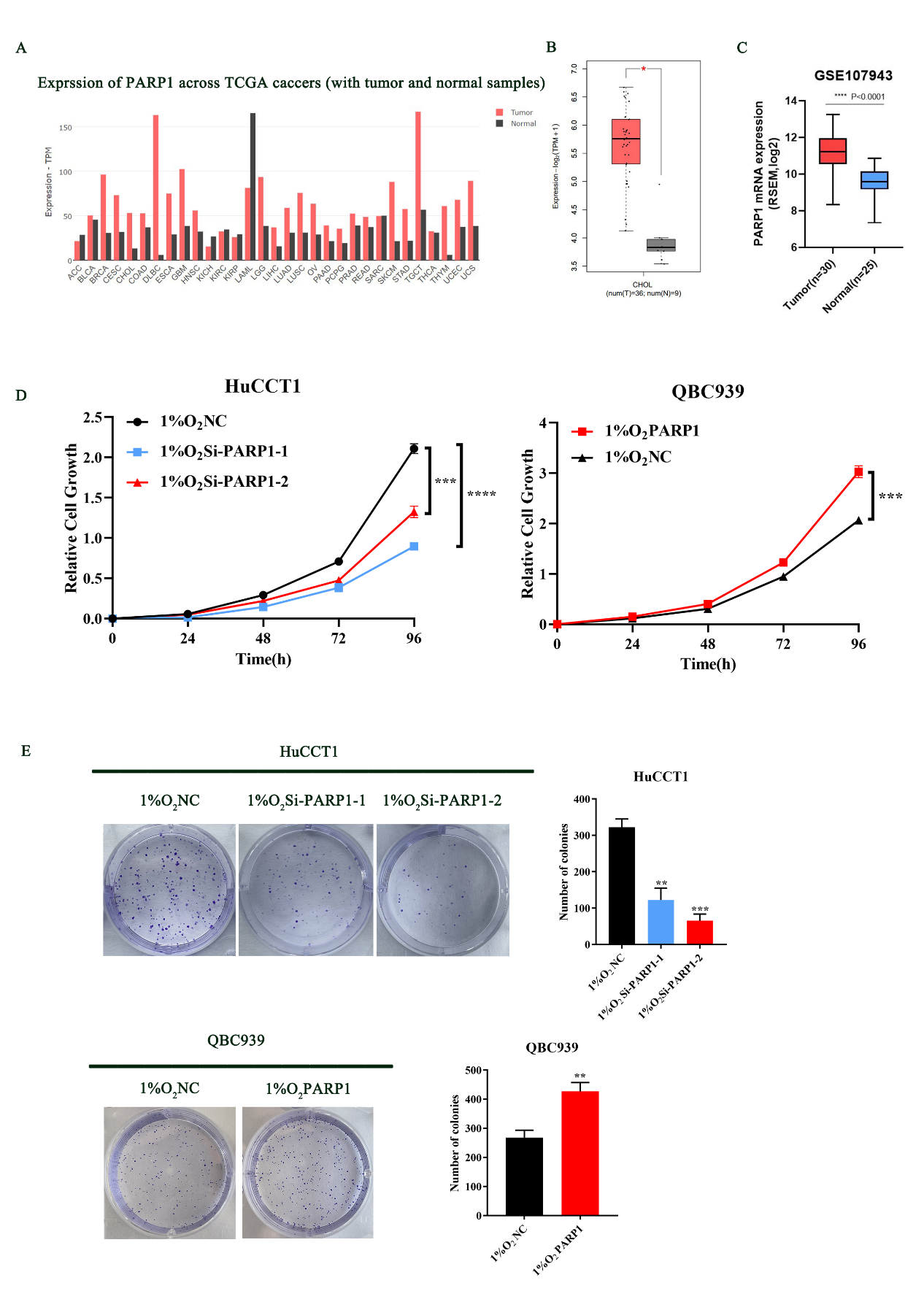


**Fig. S9.** (A-B) TCGA database showed that PARP1 was oncogenic in various of tumours including CCA. (C) CCK8 assays, and (D) Clone formation assays showed PARP1 promoted the proliferation of CCA under hypoxic conditions.

**
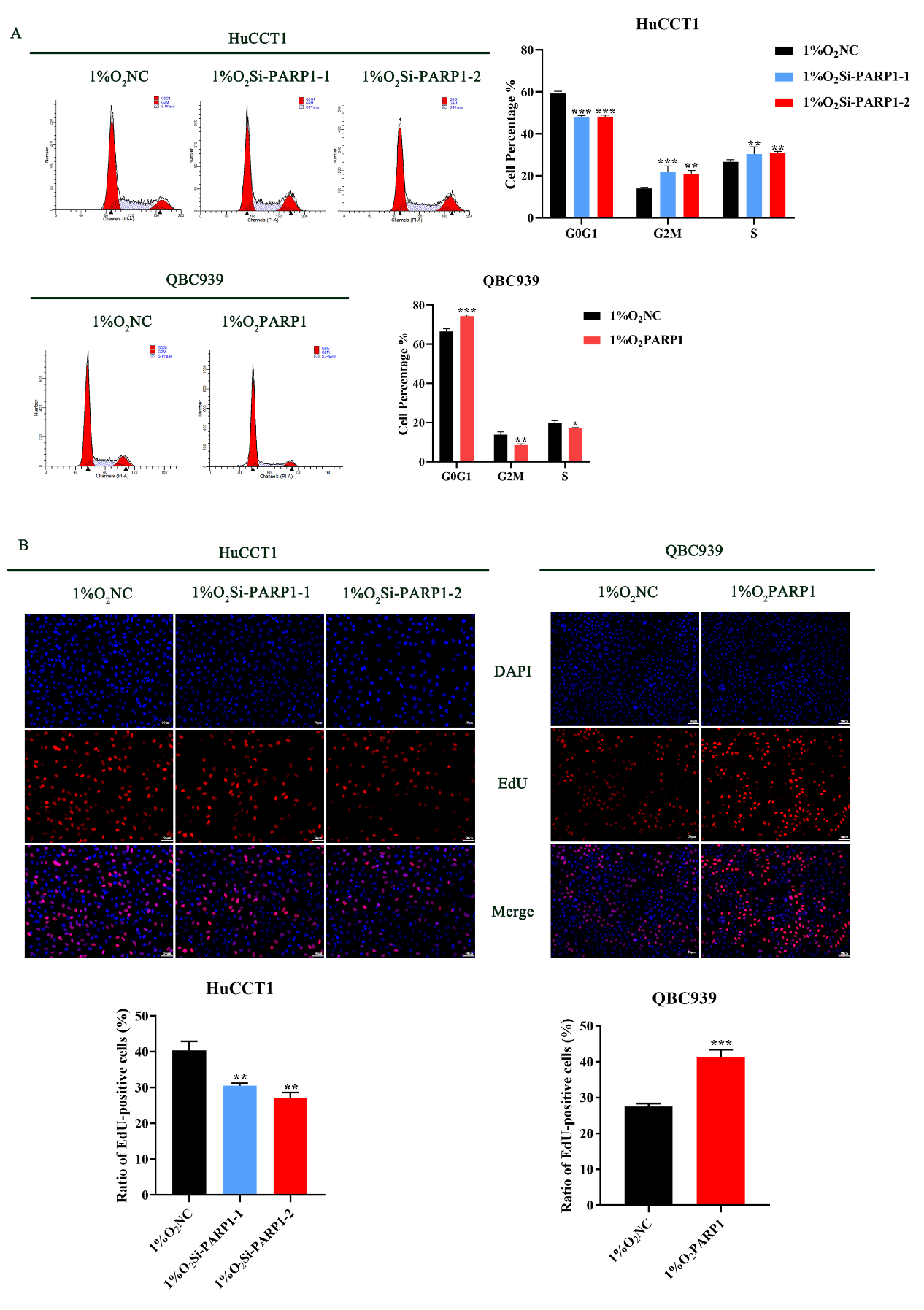
**

**Fig. S10.** (A) Cell cycle assays and (B) EdU staining assays showed PARP1 enhanced the proliferation of CCA under hypoxic conditions.

**
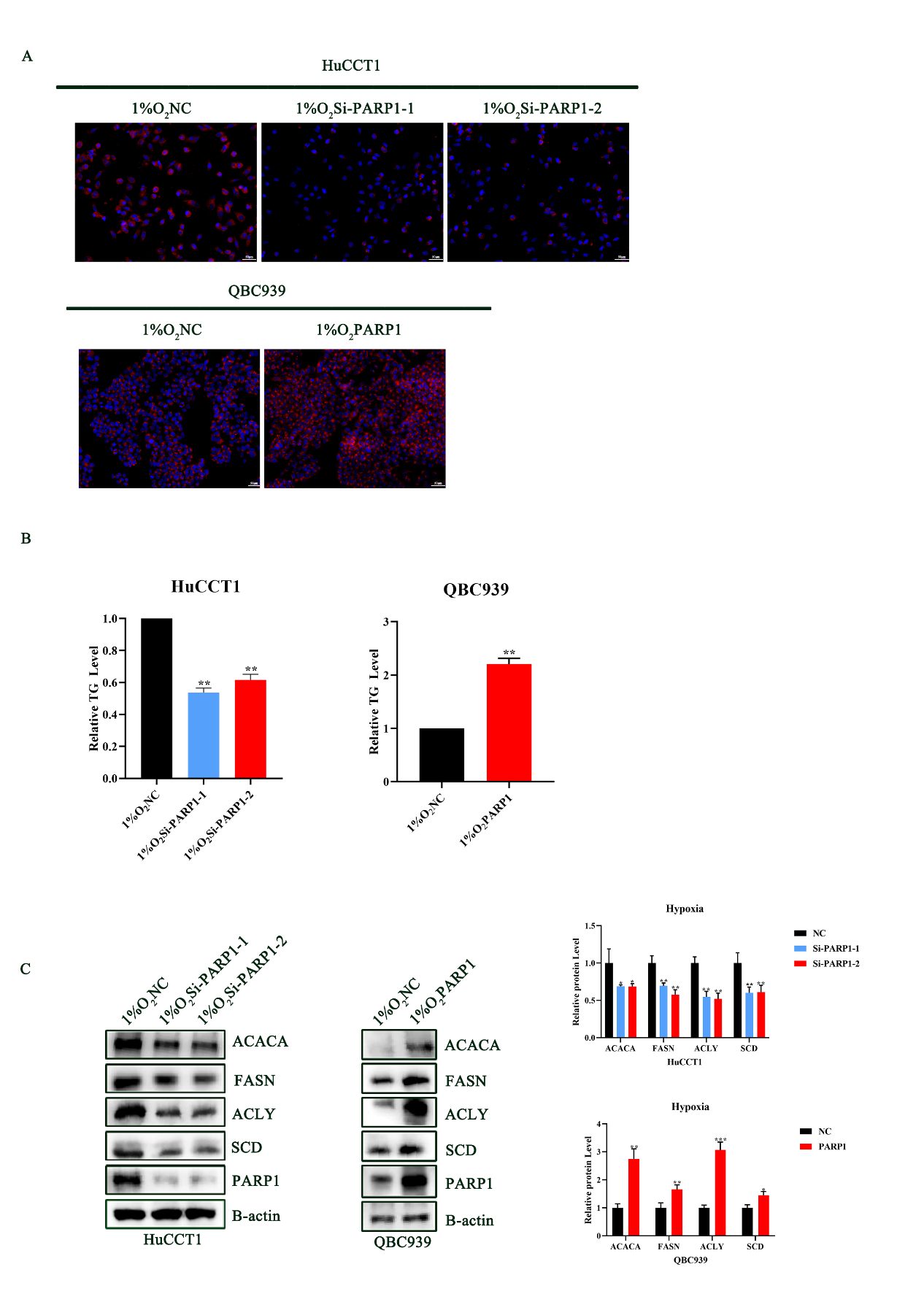
**

**Fig. S11.** (A) Nile red staining, (B) Cellular triglycerides detection, and (C) Western blot analysis showed PARP1 promoted the fatty acid synthesis of CCA under hypoxic conditions.

**
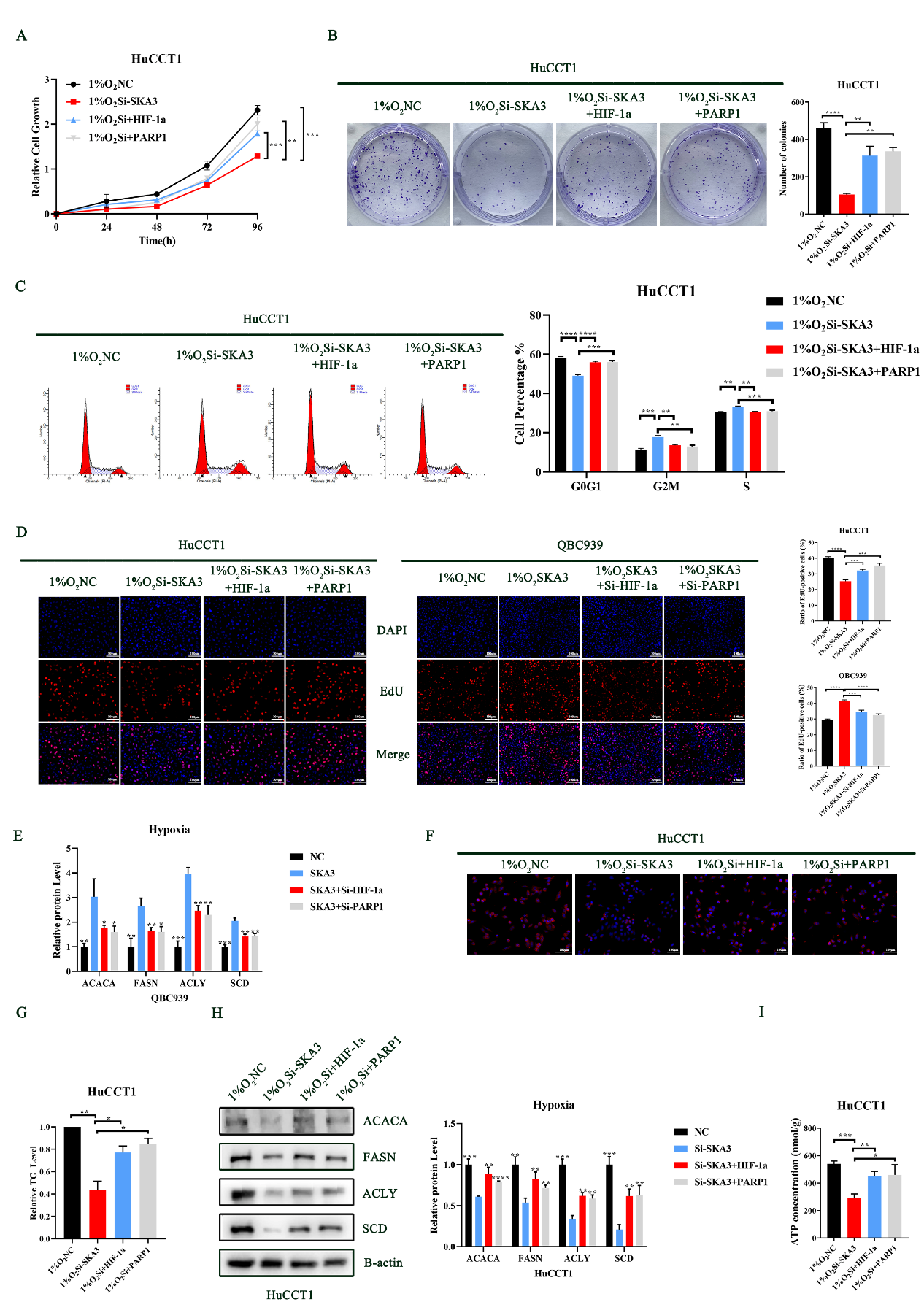
**

**Fig. S12.** (A) CCK8 assays, (B) Clone formation assays, (C) Cell cycle analysis, (D) EdU staining assays detected the proliferation of CCA cells. (E) Statistical and quantitative results of Figure 7F. (F) Nile red staining, (G) Cellular triglycerides detection, and (H) Western blot analysis showed PARP1 or HIF-1a overexpression reversed the increase of fatty acid synthesis in SKA3 Knockdown CCA cells under hypoxic conditions. (I) The ATP concentration in QBC939 cells were tested by ATP Assay Kit.
