# Supplementary material for: Hypoxia-induced SKA3 promoted cholangiocarcinoma progression and chemoresistance by enhancing fatty acid synthesis via the regulation of PAR-dependent HIF-1a deubiquitylation
Source: J Exp Clin Cancer Res. 2023 Oct 11;42:265. doi: 10.1186/s13046-023-02842-7 (PMC10565972; doi:10.1186/s13046-023-02842-7)
Supplement: Supplementary file 2 — Additional file 2. Supplementary materials and methods. [file 13046_2023_2842_MOESM2_ESM.doc]

**Supplementary materials and methods**

**1.cell culture**

Human CCA cell lines RBE, HCCC9810, QBC939, HuCCT1 and human intrahepatic biliary epithelial cells (HiBEC) were purchased from the Chinese National Human Genome Center (Shanghai, China). These cells were cultured in DMEM medium (Gibco, USA) supplemented with 10% fetal bovine serum (FBS, Gibco) and antibiotics (1% penicillin/streptomycin; Gibco). For normoxic conditions (21% oxygen), cell lines were maintained in a humidified atmosphere at 37 °C in 5% CO2 incubator and for hypoxia treatment, cells were maintained under 1% O2, 5% CO2 and 94% N2.

**2. CCK8 assay**

CCK8 assay kit (Dojindo, Japan) was used to assess cell proliferation. Cells were seeded in 96-well plates at 1×10^3^ cells/well. In each day of the subsequent 5 days, 100μ medium containing 10μ CCK8 reagent was added into each well. The absorbance at 450nm was measured with a microplate reader.

**3. Cell cycle analysis**

Flow cytometry was used to analyze the cell cycle using cell cycle staining kit (MultiSciences, China). After 48h of transfection, cells were harvested and washed with PBS and then fixed in 75% ethanol overnight at -20°C. Subsequently, cells were washed with PBS three times and incubated with propidium iodide (10mg/ml) for 30min at room temperature in the shadow. The percentage of cells in each phase was analyzed with FACS Calibur flow cytometer, Cell Quest (BD Biosciences, USA).

**4. EdU assay**

EdU cell proliferation kit (Beyotime, China) was used for EdU assay to assess cell proliferation. Cells were planted in 24-well plate at 80% confluence and cultured for 24h, followed by 2h incubation with 10μM EdU medium. The cells were fixed in 4% paraformaldehyde for 15min and permeabilized with 0.3% Triton for 15 min. Sequentially the cells were stained with Alexa Fluor 555 azide for 30 min and Hoechst 33342 for 10 min in the dark. The cells were photographed under a fluorescence microscope (Olympus, Japan).

**5. Nile red staining**

To visualize lipid droplets, cultured cells were fixed in 4% paraformaldehyde solution on the 6-well plates, washed in 1xPBS prior to staining with 5 μg/ml Nile red solution (MCE, USA) for 15 min. Samples were then washed with PBS twice, then stained with DAPI. The images were visualized by immunofluorescence microscopy.

**6. Triglyceride and cholesterol assays**

Intracellular triglyceride contents were assayed using kits purchased from NanJing JianCheng Bioengineering Institute according to the manufacturer’s recommended protocols. The values were normalized to cellular protein and the protein concentration in the resulting lysates was determined using the BCA Protein Assay Kit (Thermo Scientific, USA).

**7. Determination of ATP content**

CCA cells ATP content was determined by an Enhanced ATP Assay Kit (Beyotime, China) according to the manufacturer's instructions, and the results are shown in arbitrary units.

**8.Oil Red staining**

Frozen sections from human CAA and paired para-tumor tissues were incubated with ORO working solution (Sigma-Aldrich) at room temperature, and counterstained the sections with hematoxylin solution (Sangon, China). The red lipid droplets were visualized by microscopy.

**9. RNA extraction and RT-qPCR**

Total RNA was extracted from cell lines and tissue samples using TRIzol reagent (Invitrogen, USA) according to the manufacturer’s protocol. Reverse transcription was performed using HiScript Q-RT SuperMix (Vazyme, China). RT-qPCR was performed with AceQ qPCR SYBR Green Master Mix (Vazyme, China). RNA relative expression was calculated using 2^-△△CT^ method with B-actin as an endogenous control. The sequences of the related primers were listed below.

**10. Total protein extraction and western blot analysis**

Total protein was extracted from cell lines with NP-40 Lysis Buffer (Beyotime, China) supplemented with 1mM PMSF. The proteins were separated using SDS-PAGE electrophoresis and transferred to PVDF membranes. The membranes were blocked in 5% nonfat powered milk TBST solution for 2 hours and incubated overnight in appropriate primary antibody at 4°C. The membranes were incubated in the corresponding HRP secondary antibody for 2 hours at room temperature. The related antibodies were listed at the end of this file.

**11. Immunohistochemical staining and immunofluorescence staining**

Microarrays of cholangiocarcinoma tissues and sections of mice xenografts were prepared for immunohistochemical staining. The slides were immersed in 3% H2O2 for 5 min at room temperature to block endogenous peroxidase activity and incubated in sodium citrate buffer for 15 min at 95°C for antigen retrieval. After being blocked with 5% normal goat serum for 10 min, the slides were incubated with corresponding antibodies overnight at 4°C, followed by incubation with appropriate secondary antibody for 1 hour at room temperature. Nuclei were visualized by DAPI (Beyotime, China) staining. The images were taken through a fluorescence microscope (Olympus, Japan). For immunofluorescence, cells attached to slides were fixed with 4% paraformaldehyde and permeabilized with Immunostaining Permeabilization Buffer with Saponin (Beyotime, China). After washing, the sliders were blocked with 5% BSA in PBS for 1 hour at room temperature and then incubated with appropriate primary antibody overnight at 4°C. The cells were washed three times and incubated with corresponding secondary antibody for 1 hour at room temperature. Nuclei were stained with DAPI. The slides were photographed under a fluorescence microscope (Olympus, Japan).

**12. Forced Expression and Knockdown of Target Genes**

Small interferring RNA (siRNA) targeting SKA3, PARP1 and Hif-1a were ordered from GeneChem (Shanghai, China). The Hif-1a and PARP1 overexpression plasmids were ordered from GeneCopoeia. The cells were transfected with siRNA and plasmids using lipofectamine 3000 (Invitrogen, USA) according to the manufacturer’s instruction.The SKA3 overexpression lentivirus were ordered from Coreus Biotechnology Co., Ltd(Nanjing, China). For stable cell lines production, CCA cell lines were transfected with lentivirus for 48 h, followed by puromycin treatment (2 μg/ml, 3 days and longer). The Hif-1a and PARP1 overexpression plasmids were ordered from GeneCopoeia. The oligonucleotides sequences were listed in below.

**13.** **Chemicals**

PDD 00017273 (PARGi, MCE); P5091 (USP7i, MCE); HY-13259 (MG132. MCE); LY 188011(Gemcitabine, MCE).

**14.Animal experiment**

6-week-old male BALB/c nude mice (GemPharmatech Co. Ltd, China) were purchased for animal experiment. Suspensions containing 5.0×10^6^ QBC939 cells were subcutaneously injected into the groin region of nude mice. For gemcitabine efficacy analysis, the animals of each group (n=3) were assigned to gemcitabine (50mg/kg) twice a week when the tumors reached approximately 3mm in diameter. Every other group (n=3) received saline as a control. The tumor was measured weekly with calipers until it was removed 4 weeks later. The tumor volume was calculated by volume=width2× Length /2.

**15.Bioinformatic analysis**

Public datasets of mRNA expression data in CCA tissues were analyzed from The Cancer Genome Atlas (TCGA) database. Criteria in TCGA, GSE107943 and our Tissues was set as: Log_2_FC>3, P<0.001.

**Sequences of primers and siRNAs.**

| **1. Primers used in RT-qPCR analysis** | | | | |
| --- | --- | --- | --- | --- |
| **Gene** | | **Forward Primer** | | **Reverse Primer** |
| SKA3 | | TACACGAGCAAGAAGCCATTAAC | | GGATACGATGTACCGCTCAAGT |
| FASN | | AAGGACCTGTCTAGGTTTGATGC | | TGGCTTCATAGGTGACTTCCA |
| ACLY | | TCGGCCAAGGCAATTTCAGAG | | CGAGCATACTTGAACCGATTCT |
| ACC | | ATGTCTGGCTTGCACCTAGTA | | CCCCAAAGCGAGTAACAAATTCT |
| SCD | | TCTAGCTCCTATACCACCACCA | | TCGTCTCCAACTTATCTCCTCC |
| PARP1 | | CGGAGTCTTCGGATAAGCTCT | | TTTCCATCAAACATGGGCGAC |
| Hif-1a | | GAACGTCGAAAAGAAAAGTCTCG | | CCTTATCAAGATGCGAACTCACA |
| B-actin | | CATGTACGTTGCTATCCAGGC | | CTCCTTAATGTCACGCACGAT |
|  | |  | |  |
| **2. siRNAs** | | | | |
| **Gene** | **Sense (5’-3’)** | |  | |
| SKA3#1 | GGAAGAGCCCGUAAUUGUA | |  | |
| SKA3#2 | GAUCGUACUUCGUUGGUUU | |  | |
| SKA3#3 | AAUCCAGGCUCAAUGAUAA | |  | |
| PARP1#1 | CGACCUGAUCUGGAACAUCAA | |  | |
| PARP1#2 | CGCAGCUUCAUAACCGAAGAUU | |  | |
| Hif-1a#1 | CAUGAAAGCACAGAUGAAUTT | |  | |
| Hif-1a#2 | GAUUAACUCAGUUUGAACUTT | |  | |
| Hif-1a#3 | CCACAUUCACGUAUAUGAUTT | |  | |
| FASN | TGGAGCGTATCTGTGAGAA | |  | |
| ACLY | GACCAAAGATGGAGTCTAT | |  | |
| SCD | ATATCAGGGCGAATGTCGTCT | |  | |
| ACACA | GCUCAUACACUUCUGAAUATT | |  | |
|  |  | |  | |

**Primary antibodies used in this study.**

| **Antibody** | **Company (Cat. No.)** | **Working Concentration Dilutions** |
| --- | --- | --- |
| SKA3 | Abcam (ab186003) | WB: 1/2000 IP:2ug/mg |
| SKA3 | Sino Biological  (203255-TTTTTT44) | WB: 1/1000 |
|  | (203255-T44) | IP: 2ul/mg IHC:1/100 |
| SKA3 | Bioss (bs-7848R) | IHC:1/100 IF:1/100 |
| FASN | Proteintech (10624-2-AP) | WB: 1/1000 |
| ACLY | Abcam (ab40793) | WB: 1/2000 |
| ACC | Abcam (ab45174) | WB: 1/1000 |
| SCD | Abcam (ab236868) | WB: 1/1000 |
| HIF-1a | Abcam (ab51608) | WB: 1/1000 IHC:1/100 |
| PARP1 | Proteintech (13371-1-AP) | WB: 1/1000 IHC:1/50 |
|  |  | IF:1/50 |
| PAR | R&D Systems (4335-MC-100) | WB: 1/1000 |
| USP7 | Proteintech (66514-1-Ig) | WB: 1/5000 IP:2ug/mg |
| Ki-67 | Proteintech (27309-1-AP) | IHC:1/100 |
| Gamma H2A.X | Abcam (ab81299) | IF：1/250 |
| B-actin | Proteintech (20536-1-AP) | WB: 1/1000 |
|  |  |  |
