# Supplementary material for: Hypoxia-induced SKA3 promoted cholangiocarcinoma progression and chemoresistance by enhancing fatty acid synthesis via the regulation of PAR-dependent HIF-1a deubiquitylation
Source: J Exp Clin Cancer Res. 2023 Oct 11;42:265. doi: 10.1186/s13046-023-02842-7 (PMC10565972; doi:10.1186/s13046-023-02842-7)

**Supplementary Table. 1** Association of SKA3 expression with clinicopathologic features of CCA.


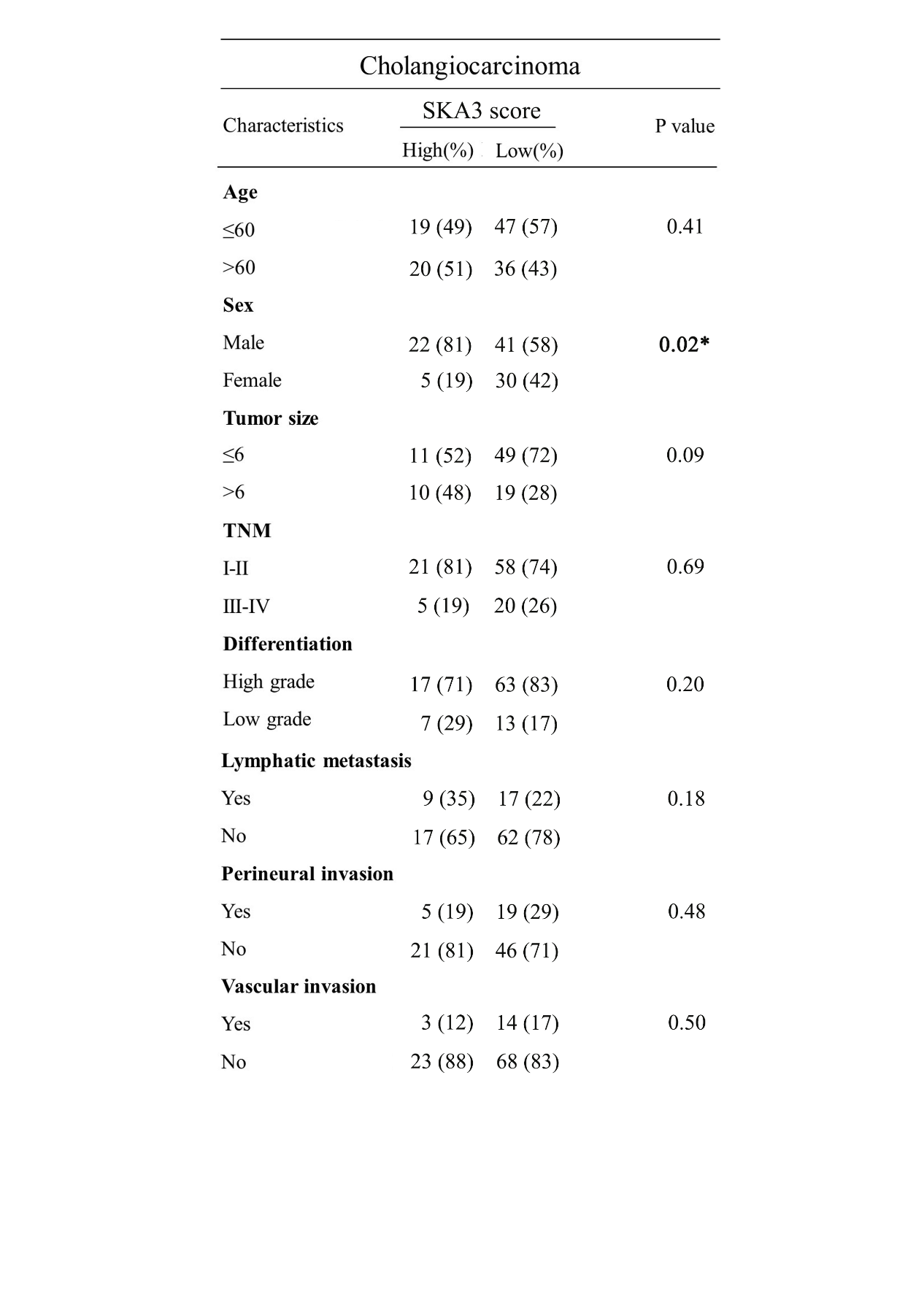


**Supplementary Table. 2** Association of HIF-1a expression with clinicopathologic features of CCA.


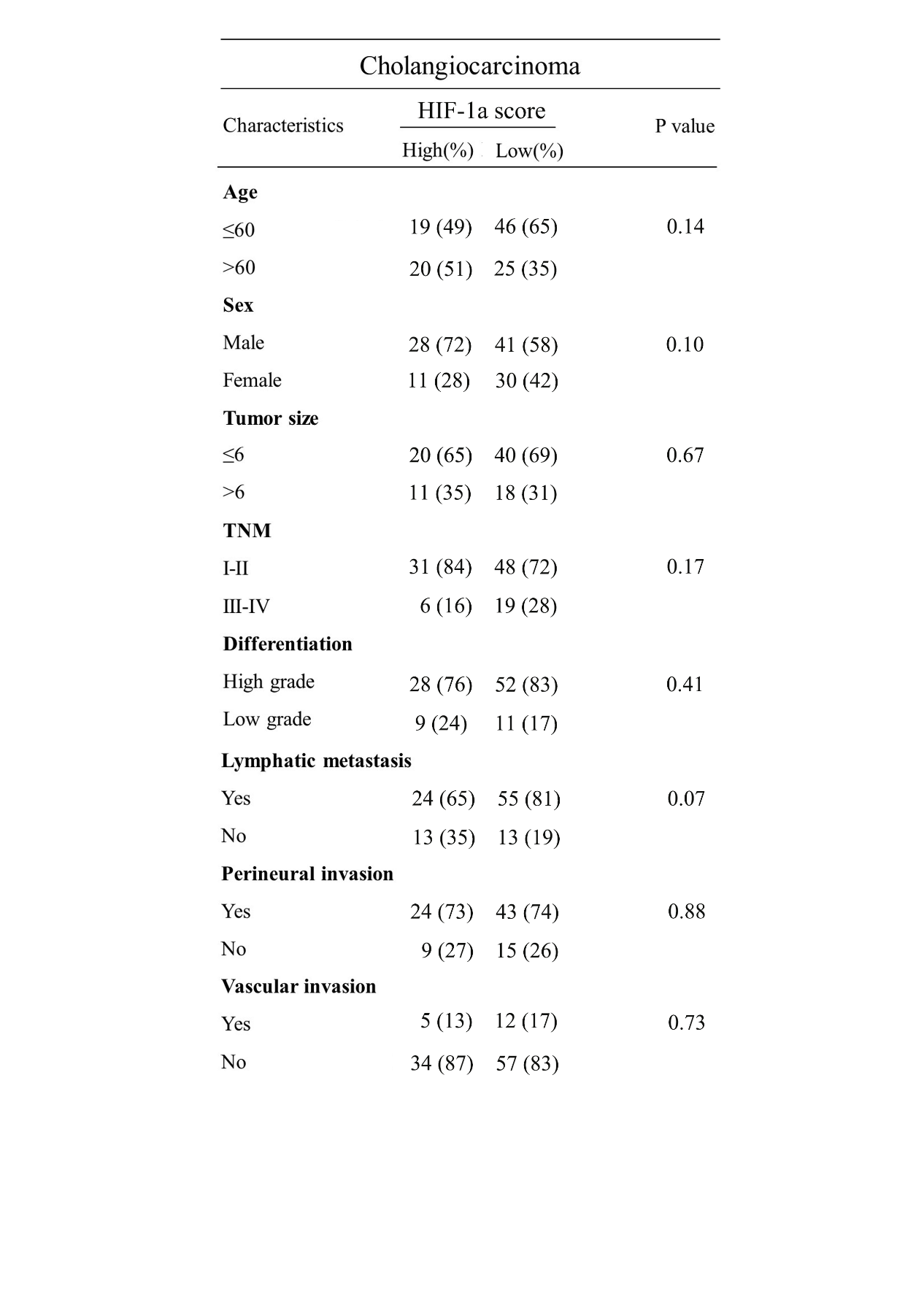

Supplement: Supplementary file 3 — Additional file 3: Supplementary Table 1. Association of SKA3 expression with clinicopathologic features of CCA. Supplementary Table 2. Association of HIF-1a expression with clinicopathologic features of CCA. [file 13046_2023_2842_MOESM3_ESM.docx]
